# Lysine crotonylation of DgTIL1 at K72 modulates cold tolerance by enhancing DgnsLTP stability in chrysanthemum

**DOI:** 10.1111/pbi.13533

**Published:** 2021-01-21

**Authors:** Qiuxiang Huang, Xiaoqin Liao, Xiaohan Yang, Yunchen Luo, Ping Lin, Qinhan Zeng, Huiru Bai, Beibei Jiang, Yuanzhi Pan, Fan Zhang, Lei Zhang, Yin Jia, Qinglin Liu

**Affiliations:** ^1^ Department of Ornamental Horticulture Sichuan Agricultural University Chengdu China

**Keywords:** temperature‐induced lipocalins, nonspecific lipid transfer proteins, cold stress, protein interaction, lysine crotonylation, peroxidase

## Abstract

Lysine crotonylation of proteins is a recently identified post‐translational modification (PTM) in plants. However, the function of lysine‐crotonylated proteins in response to abiotic stress in plants has not been reported. In this study, we identified a temperature‐induced lipocalin‐1‐like gene (*DgTIL1*) from chrysanthemum and showed that it was notably induced in response to cold stress. Overexpression of *DgTIL1* enhanced cold tolerance in transgenic chrysanthemum. Ubiquitin membrane yeast two‐hybrid (MYTH) system and bimolecular fluorescence complementation (BIFC) assays showed that DgTIL1 interacts with a nonspecific lipid transfer protein (DgnsLTP), which can promote peroxidase (*POD*) gene expression and POD activity to reduce the accumulation of reactive oxygen species (ROS) and improve resistance to cold stress in *DgnsLTP* transgenic chrysanthemum. In addition, we found that DgTIL1 was lysine crotonylated at K72 in response to low temperature in chrysanthemum. Moreover, lysine crotonylation of DgTIL1 prevented DgnsLTP protein degradation in tobacco and chrysanthemum. Inhibition of DgnsLTP degradation by lysine crotonylation of DgTIL1 further enhanced *POD* expression and POD activity, reduced the accumulation of ROS under cold stress in *DgTIL1* transgenic chrysanthemum, thus promoting the cold resistance of chrysanthemum.

## Introduction

Low temperature markedly impairs the growth and development of plants; however, plants have corresponding defence systems to prevent damage from low temperatures. Cold regulatory proteins (CORs), dehydration‐responsive element (DRE)‐binding protein (DREB) transcription factors and antifreeze molecules maintain the stability of the plasma membrane and reduce the toxicity of reactive oxygen species (ROS) (Krasensky and Jonak, 2012; Miura and Furumoto, 2013). To protect against low temperature stress, post‐translational modifications (PTMs) can regulate cold‐responsive genes, such as the ubiquitination of BT2, which enhances the cold resistance of MdMYB23 in apple (An *et al*., 2018), and phosphorylation of inducer of CBF expression 1 (ICE1), which regulates the stability of ICE1 and freezing tolerance in *Arabidopsis* (Li *et al*., 2017; Zhao *et al*., 2017). Lysine crotonylation, which was identified in 2011 (Tan *et al*., 2011), is a novel PTM. According to recent reports in tobacco, papaya, tea plants and rice (Sun *et al*., 2017; Xu *et al*., 2017; Liu *et al*., 2018a,b; Lu *et al*., 2018; Sun *et al*., 2019), lysine crotonylation of histones is related to signal transduction and cellular physiology and mainly participates in the process of protein biosynthesis, folding and degradation, chromatin organization, carbon metabolism and photosynthesis. However, the function of lysine‐crotonylated proteins in response to abiotic stress has not been reported.

Temperature‐induced lipocalins (TILs) are members of the lipocalin family, and experiments have shown that TILs are located on the plasma membrane (Charron *et al*., 2005) and play an important role in increasing the stability of the plasma membrane to improve the cold resistance of *Arabidopsis thaliana* (Uemura *et al*., 2006). TIL proteins were first identified in *A. thaliana (AtTIL*) and wheat (*TaTIL*), and their transcripts were up‐regulated during cold acclimation as determined by Northern Blot analysis (Charron *et al*., 2002). In addition, the transcript level of *TaTIL* was higher, and the protein level of TaTIL was significantly higher in low temperature‐treated wheat than in the control (Kawamura and Uemura, 2003). Furthermore, overexpression of *MfTIL1* can also mediate the up‐regulation of the transcript level of cold‐responsive genes, such as the CBF transcription factor and COR15a in *Medicago falcate* to improve the cold tolerance of plants (He *et al*., 2005). However, the underlying molecular regulatory mechanism of *TIL1* is unclear and information on *TILs* has not been reported in chrysanthemum.

Nonspecific lipid transfer proteins (nsLTPs), which are localized on the plasma membrane, are widely present in multiple organisms (Debono *et al*., 2009) and harbour an eight‐cysteine (c) motif backbone, which forms four disulphide bonds that facilitate binding between different lipids and hydrophobic compounds (Kader, 1996; Yeats and Rose, 2008) and protect plants from adverse environmental conditions. Plant *nsLTP* can respond to cold stress, such as by promoting the overexpression of *AtLTP3*, which reduces electrolyte leakage induced by cold stress to improve soluble sugar accumulation and the survival rate of *A*. *thaliana* (Debono *et al*., 2009), and inducing the expression of *OsLTPL159*, which has been shown to regulate the activity of POD enzymes in rice to improve plant cold tolerance (Zhao *et al*., 2020). However, the TIL‐mediated molecular regulatory mechanism of nsLTP has not yet been studied and the biological function of nsLTPs has not been reported in chrysanthemum.

In our study, we found that *DgTIL1* acts as a regulatory gene under low temperature stress and *DgTIL1* overexpression improves the cold resistance of chrysanthemum. Ubiquitin membrane yeast two‐hybrid (MYTH) system and bimolecular fluorescence complementation (BIFC) assays showed that DgTIL1 interacts with DgnsLTP and *DgnsLTP* overexpression can promote *POD* expression and POD activity to reduce the accumulation of ROS and improve resistance to cold stress in *DgnsLTP* transgenic chrysanthemum. Lysine crotonylation of DgTIL1 enhances the interaction between DgTIL1 and DgnsLTP in tobacco and prevents DgnsLTP protein degradation in tobacco and chrysanthemum. Finally, inhibition of degradation of DgnsLTP by lysine crotonylation of DgTIL1 further enhances POD activity and minimizes ROS in *DgTIL1* transgenic chrysanthemum.

## Results

### *DgTIL1* is responsive to low temperature

To identify cold‐responsive *TIL* genes in chrysanthemum, we performed transcriptome analyses (accession number GSE117262) using cold‐treated (4°C for 24 h and −4°C for 4 h) and non‐treated (temperature, 25°C) chrysanthemum seedlings. The results showed that 4 *TIL* genes were significantly induced at the transcription level by cold treatment (Table S1). Among these genes, *DgTIL1* (log2fold change = 4.2) (GenBank accession number: MT219513) was notably induced via cold treatment. Therefore, *DgTIL1* was chosen for further investigation.

**Figure 1:**
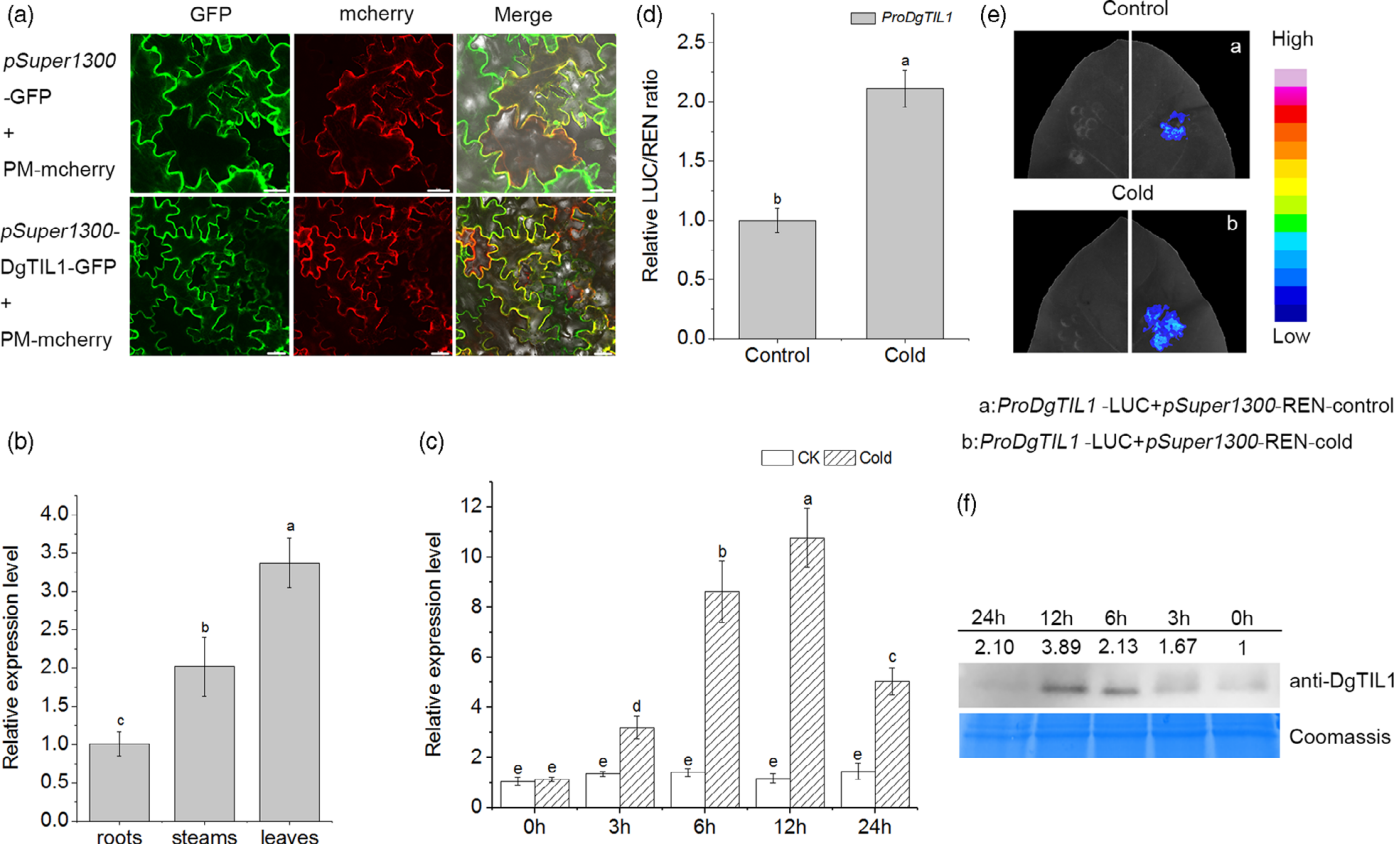
*DgTIL1* is responsive to low temperature. (a) Subcellular localization of *pSuper1300*‐DgTIL1‐GFP in tobacco leaves. *pSuper1300*‐GFP and CD3‐1007 (an mCherry‐labelled plasma membrane marker) were used as negative controls. Scale bars, 20 μm. (b) Expression of *DgTIL1* in the roots, stems, and leaves of WT chrysanthemum at normal temperature using qRT‐PCR (*P *< 0.05) (Data represent means and standard errors of 3 replicates, 20 plants per replicate). (c) Relative expression levels of the *DgTIL1* gene in the WT with low temperature treatment (4°C). CK represents the control under control conditions (25°C day/22°C night). (d and e) DLA assay and LCI assay of the native promoter of *ProDgTIL1* after transient expression in tobacco, and the control (25°C day/22°C night) and cold (4°C for 12 h) treatment results were used for comparison. (f) Immunoblot analysis of DgTIL1. Proteins from WT chrysanthemum leaves under low temperature (4°C) treatment were probed with anti‐DgTIL1 (1:1000, from PTM BIO, Hangzhou, China), and Coomassie blue staining was used to demonstrate consistent protein loading.

The full‐length cDNA of *DgTIL1* contains 558 bp and encodes a predicted protein of 186 amino acids. Alignment of the TILs amino acid sequences of various plants demonstrated that DgTIL1 contains three structurally conserved region (SCR) (Figure S1a).The phylogenetic analysis demonstrated that DgTIL1 is highly homologous with the temperature‐induced lipocalin‐1‐like protein and closely related to TcTIL1 from *Tanacetum cinerariifolium* (Figure S1b).To determine the subcellular localization of DgTIL1, *pSuper1300‐DgTIL1‐GFP* and the plasma membrane marker protein *PM‐mCherry* (CD3‐1007) were coexpressed in the epidermal cells of tobacco leaves, and the results showed that DgTIL1 was localized in the plasma membrane (Figure 1a).

We measured the expression pattern of *DgTIL1* in different tissues and at different times at low temperature in the WT leaves by qRT‐PCR and found that the transcript abundance of *DgTIL1* in the leaves was significantly higher (*P *< 0.05) than that in the roots and stems (Figure 1b). In addition, the expression of *DgTIL1* in the leaves reached the highest value (*P *< 0.05) after 12 h of low temperature treatment (Figure 1c). The promoters (1.3 kbp) of the *DgTIL1* and *LUC* reporter genes were inserted into *pSuper1300* to probe the function of the natural promoter. We found that the luciferase (LUC) activity of *DgTIL1* natural promoter was enhanced at low temperatures as detected by a dual luciferase reporter gene assay (DLA) (Figure 1d), and the luciferase complementation imaging (LCI) experiments also showed the same results (Figure 1e), indicating that low temperature activates the transcription of *DgTIL1*.

We performed proteomics sequencing and found that the protein expression of DgTIL1 was 1.20‐times higher in chrysanthemum under low temperature conditions than the control chrysanthemum. The mass spectrometry proteomics data were deposited into the ProteomeXchange consortium via the PRIDE partner repository, with the dataset identifier PXD010297. In addition, a gel blot analysis showed that the protein expression generally gradually increased with increasing low temperature treatment time and the protein abundance peaked at 12 h as evidenced by an anti‐DgTIL1 antibody (Figure 1f). These results revealed that DgTIL1 responds to low temperature.

### Overexpression of *DgTIL1* improves the cold resistance of chrysanthemum

**Figure 2:**
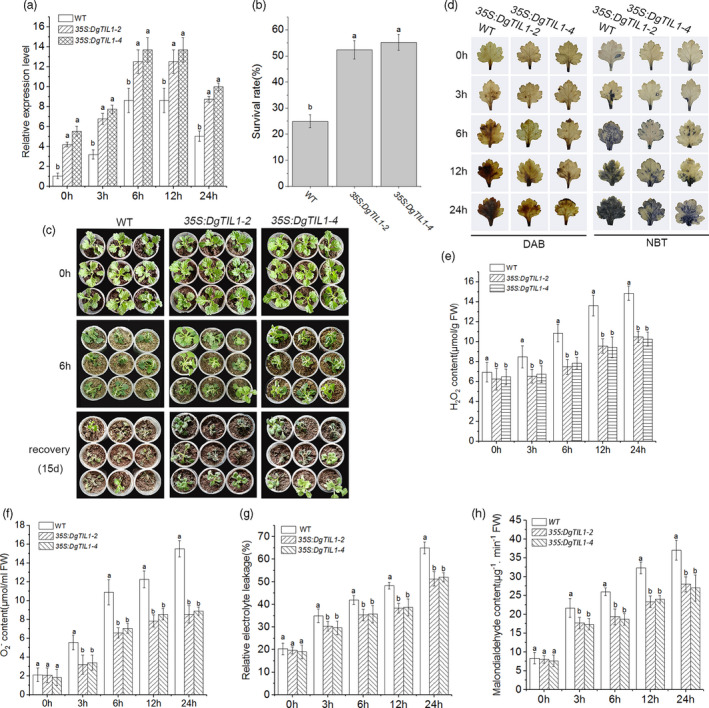
Less ROS products in *DgTIL1* transgenic chrysanthemum. (a) Expression levels of *DgTIL1* in the WT and transgenic chrysanthemum under control conditions (25°C day/22°C night) and low temperature stress at 4°C (*P *< 0.05). (b) Survival rates of WT and DgTIL1 transgenic chrysanthemum after 15 days of recovery under control conditions (25°C day/22°C night), with 3 replicates and 50 plants per replicate. (c) Chrysanthemum phenotype changes under low temperature stress at −6°C. (d) DAB and NBT staining of WT and *DgTIL1* transgenic chrysanthemum under low temperature stress at 4°C. (e and f) Comparison of accumulation of H_2_O_2_ (e) and O_2_^‐^ (f) in plant lines (WT and 35S transgenic lines) at each time point under 4°C treatment (3 replicates, 50 plants per replicate). (g and h) Analysis of relative electrolyte leakage (g) and malondialdehyde (h) in the WT and *DgTIL1* transgenic lines at each time point under 4°C treatment (3 replicates, with 50 plants per replicate).

To verify the role of *DgTIL1* in coping with cold stress, the *35S*::*DgTIL1* overexpression vector was transformed into WT chrysanthemum. Finally, we obtained four independent transgenic chrysanthemum lines and two overexpression lines (*35S::DgTIL1‐2* and *35S::DgTIL1‐4*) that exhibited relatively high expression, which were used in subsequent experiments. The qRT‐PCR experiment showed that the expression of *DgTIL1* in the OE‐2 and OE‐4 lines was significantly (*P *< 0.05) higher than that in the WT line under the cold treatment (Figure 2a).

Then, we further tested the cold resistance of the transgenic lines, and the results showed that the OE‐2 and OE‐4 lines had higher survival rates than the WT line (Figure 2b,c). Moreover, to more intuitively observe H_2_O_2_ and O_2_^‐^ accumulation under cold stress, we stained chrysanthemum leaves sampled from the transgenic and WT lines with diaminobenzidine tetrahydrochloride (DAB) and nitroblue tetrazolium (NBT) (Figure 2d). The results showed fewer brown or blue products of oxidative damage in the transgenic lines than the WT lines, and a quantitative analysis of the H_2_O_2_ and O_2_^‐^ contents showed the same results (Figure 2e,f). Moreover, the electrolyte leakage and malondialdehyde content of *DgTIL1* transgenic lines were both lower than those of the WT line (Figure 2g,h), indicating that the *DgTIL1* transgenic lines had less membrane damage than the WT line. These results suggested that the *DgTIL1* transgenic lines had less ROS accumulation than the WT lines under cold stress, which proved that *DgTIL1* overexpression can improve the cold resistance of chrysanthemum.

### DgTIL1 interacts with DgnsLTP

We screened potential DgTIL1 interaction factors in chrysanthemum with the ubiquitin MYTH system. According to the results of a BLAST search of positive clones verified by one‐to‐one yeast interaction in GenBank, we identified 10 potential interacting proteins (Table S2). Furthermore, nonspecific lipid transfer protein‐like gene was identified and named *DgnsLTP* (GenBank accession number MT192757) (Figure S2a).

**Figure 3:**
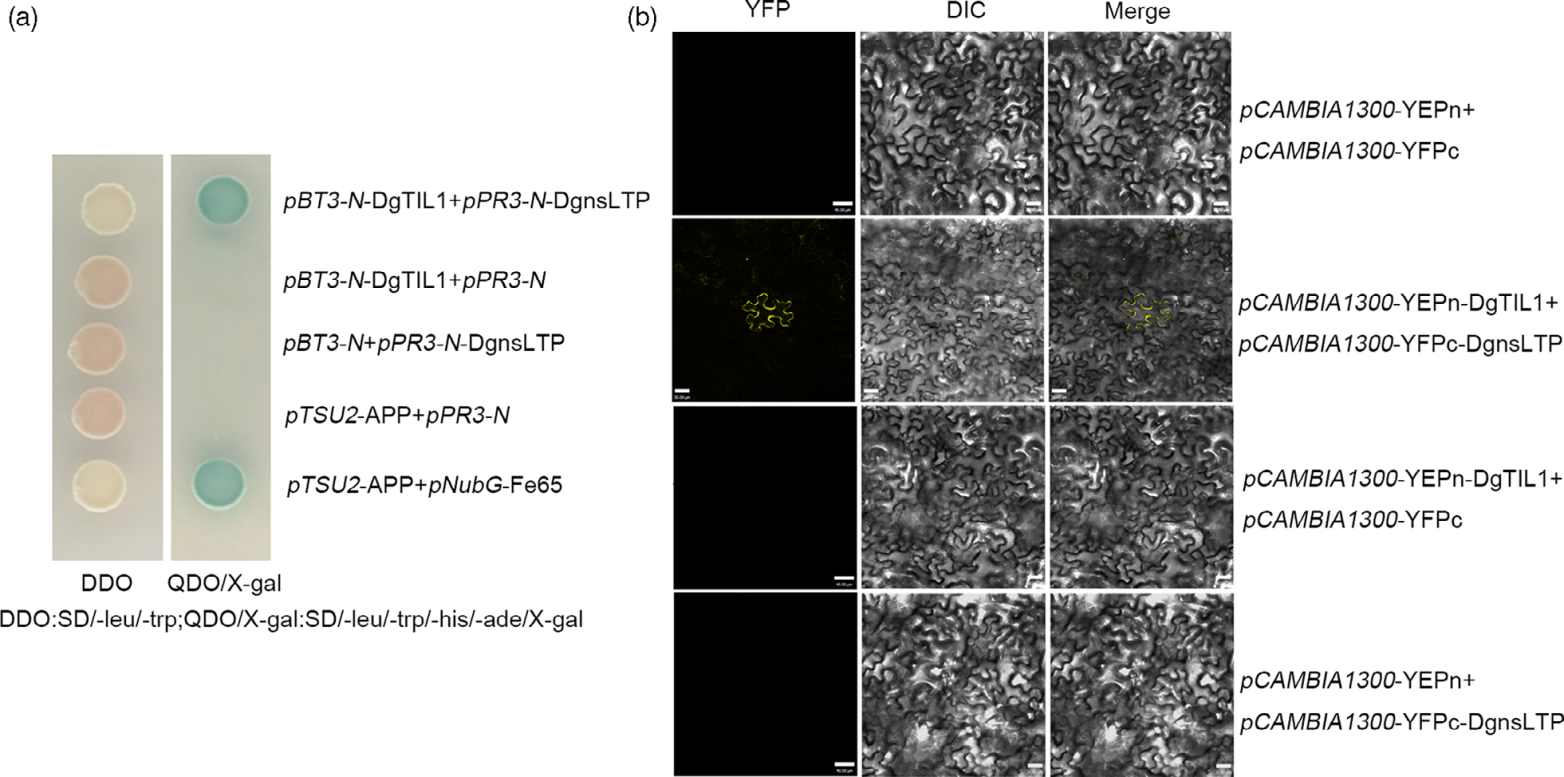
DgTIL1 interacts with DgnsLTP. (a) Split‐ubiquitin Y2H assay validating the interaction between DgTIL1 and DgLTP. *pBT3‐N*‐DgTIL1 with *pPR3‐N*, *pBT3‐N* with *pPR3‐N*‐DgnsLTP and *pTSU2*‐APP with *pPR3‐N* were used as negative controls. DDO:SD/‐leu/‐trp; QDO/X‐gal: SD/‐leu/‐trp/‐his/‐ade/X‐gal. (b) BIFC revealing the interaction of DgTIL1 with DgnsLTP in tobacco. *pCAMBIA1300‐35S‐*YFPn with *pCAMBIA1300‐35S*‐YFPc*, pCAMBIA1300‐35S*‐YFPn‐DgTIL1 with *pCAMBIA1300‐35S*‐YFPc, and *pCAMBIA1300‐35S*‐YFPn, with *pCAMBIA1300‐35S*‐YFPc‐DgnsLTP used as a negative control. Scale bars, 30 μm.

To further verify the interaction between DgTIL1 and DgnsLTP, a split‐ubiquitin yeast two‐hybrid (Y2H) assay was performed with full‐length DgTIL1 and DgnsLTP (Figure 3a). We found that pBT3‐N‐DgTIL1 interacted with pPR3‐N‐DgnsLTP. In BIFC experiments (Figure 3b), a yellow fluorescence signal was observed in the plasma membrane of cells coexpressing *DgTIL1‐YFPn* and *DgnsLTP‐YFPc*, indicating that DgTIL1 interacted with DgnsLTP on the plasma membrane.

### Overexpression of *DgnsLTP* enhanced tolerance to cold stress

**Figure 4:**
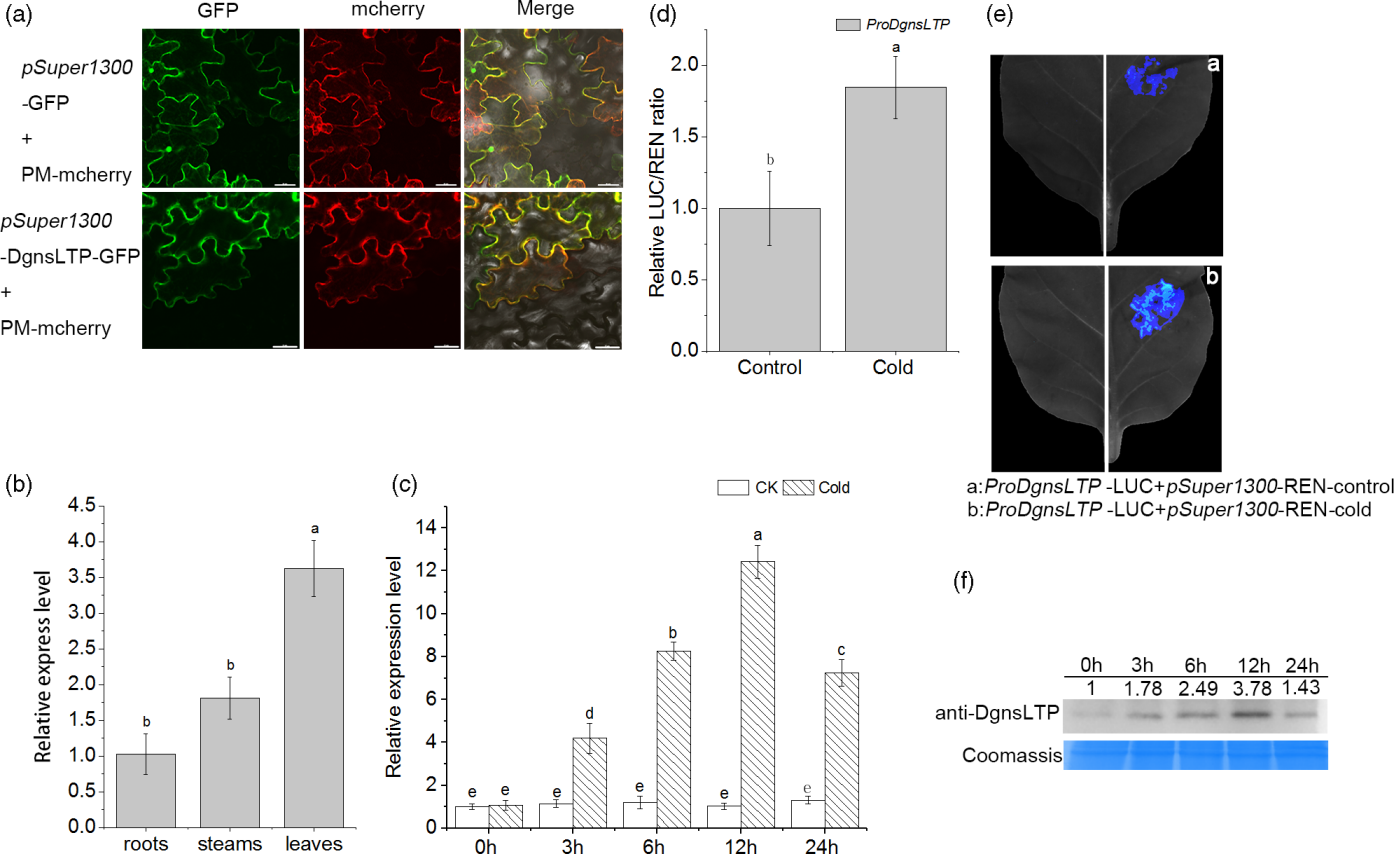
*DgnsLTP* is responsive to low temperature. (a) *pSuper1300*‐DgnsLTP‐GFP fusion protein and CD3‐1007 colocalized to the plasma membrane in tobacco cells. Scale bars, μm. (b) Expression of *DgnsLTP* in the in various tissues of WT chrysanthemum at normal temperature using qRT‐PCR (*P *< 0.05). (c) Relative expression levels of the *DgnsLTP* gene in the WT with low temperature treatment (4°C). CK represents the control under control conditions (25°C day/22°C night) (*P *< 0.05). (d and e) Analysis of native promoter of *DgnsLTP* with DLA (d) and LCI (e) assay after transient coexpression indicated proteins in tobacco under the control (25°C day/22°C night) and cold (4°C for 12 h) treatment. (f) Proteins of DgnsLTP from WT chrysanthemum leaves under low temperature (4°C) treatment were probed with anti‐DgnsLTP (1:1000, from PTM BIO) using an immunoblot analysis, and Coomassie blue staining was used to demonstrate consistent protein loading.

The full‐length cDNA of *DgnsLTP* contains 537 bp and encodes a predicted protein of 179 amino acids. Alignment of DgnsLTP amino acid sequences in various plants showed that DgnsLTP possesses a conserved eight‐cysteine motif specific to the plant LTP family (Figure S2b). The results of a subcellular localization experiment (Figure 4a) revealed that DgnsLTP is localized on the plasma membrane as evidenced by the overlapping green fluorescence of *pSuper1300‐DgnsLTP‐GFP* and red fluorescence of a plasma membrane marker (CD3‐1007). The qRT‐PCR revealed that the expression of *DgnsLTP* was higher (*P *< 0.05) in the WT chrysanthemum leaves than in other tissues (Figure 4b), and we found the *DgnsLTP* transcript level was induced by cold treatment (Figure 4c). The promoter (1.5 kbp) of *DgnsLTP* was assayed by DLA and LCI (Figure 4d,e), and we found that the LUC activity of natural *DgnsLTP* promoters was increased under cold stress. In addition, Western blot experiments were performed to verify that the DgnsLTP protein in the WT lines was induced by cold treatment using an anti‐DgnsLTP antibody (Figure 4f).

**Figure 5:**
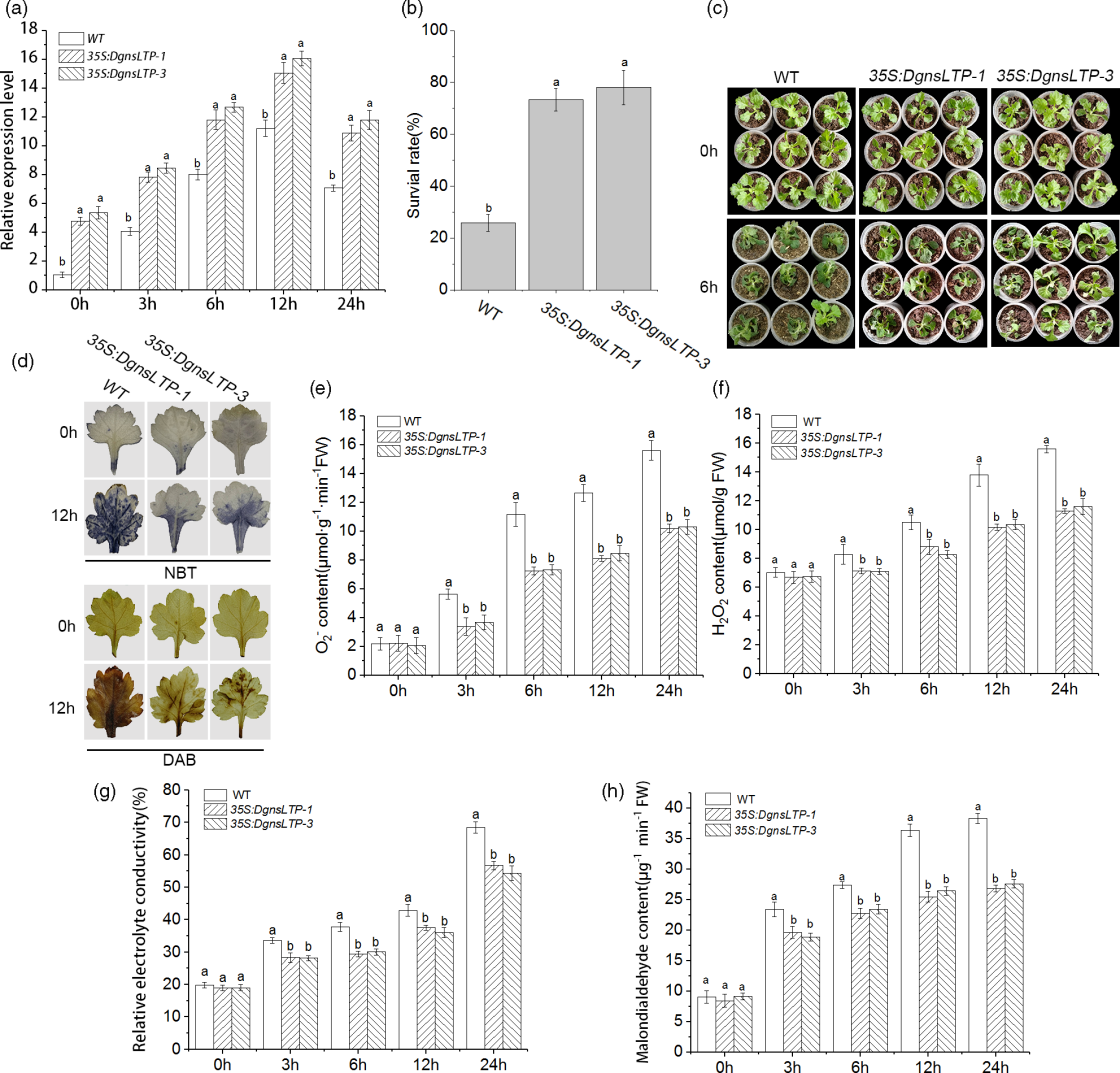
Overexpression of *DgnsLTP* enhanced cold tolerance. (a) Comparison of the relative expression of *DgnsLTP* in transgenic chrysanthemum and the WT with increasing low temperature treatment time through qRT‐PCR (*P *< 0.05). (b) Comparison of the survival rate in the WT and *DgnsLTP* transgenic lines (3 replicates, 50 plants per replicate) after 15 days of recovery under control conditions (25°C day/22°C night). (c) Phenotype comparison of *DgnsLTP* transgenic lines and WT. (d) NBT and DAB histochemical staining in the WT and *DgnsLTP* transgenic lines under low temperature stress (4°C). (e and f) Accumulation of O_2_^‐^ and H_2_O_2_ were evaluated by quantitative analysis, with 3 replicates and 50 plants per replicate. (g and h) Analysis of changes in relative electrolyte leakage (g) and malondialdehyde (h) under low temperature stress.

To determine the function of *DgnsLTP* under cold stress, two *DgnsLTP* overexpression lines (OE‐1 and OE‐3) and WT chrysanthemum were treated with or without cold. The cold‐induced transcript level of *DgnsLTP* in the OE‐1 and OE‐3 lines was higher than that in the WT lines as evaluated by qRT‐PCR assay (Figure 5a). Additionally, the survival rate was higher (Figure 5b,c), and ROS accumulation was lower in the OE‐1 and OE‐3 lines than in the WT lines based on the quantitative analysis of H_2_O_2_ and O_2_^‐^ contents and histochemical staining with DAB and NBT (Figure 5d–f). Moreover, the two transgenic lines had lower relative electrolyte leakage and less malondialdehyde than the WT control line (Figure 5g,h), indicating that *DgnsLTP* is an active regulatory gene that responds to cold stress in chrysanthemum.

### Cold induces lysine crotonylation of DgTIL1 at the K72 site

**Figure 6:**
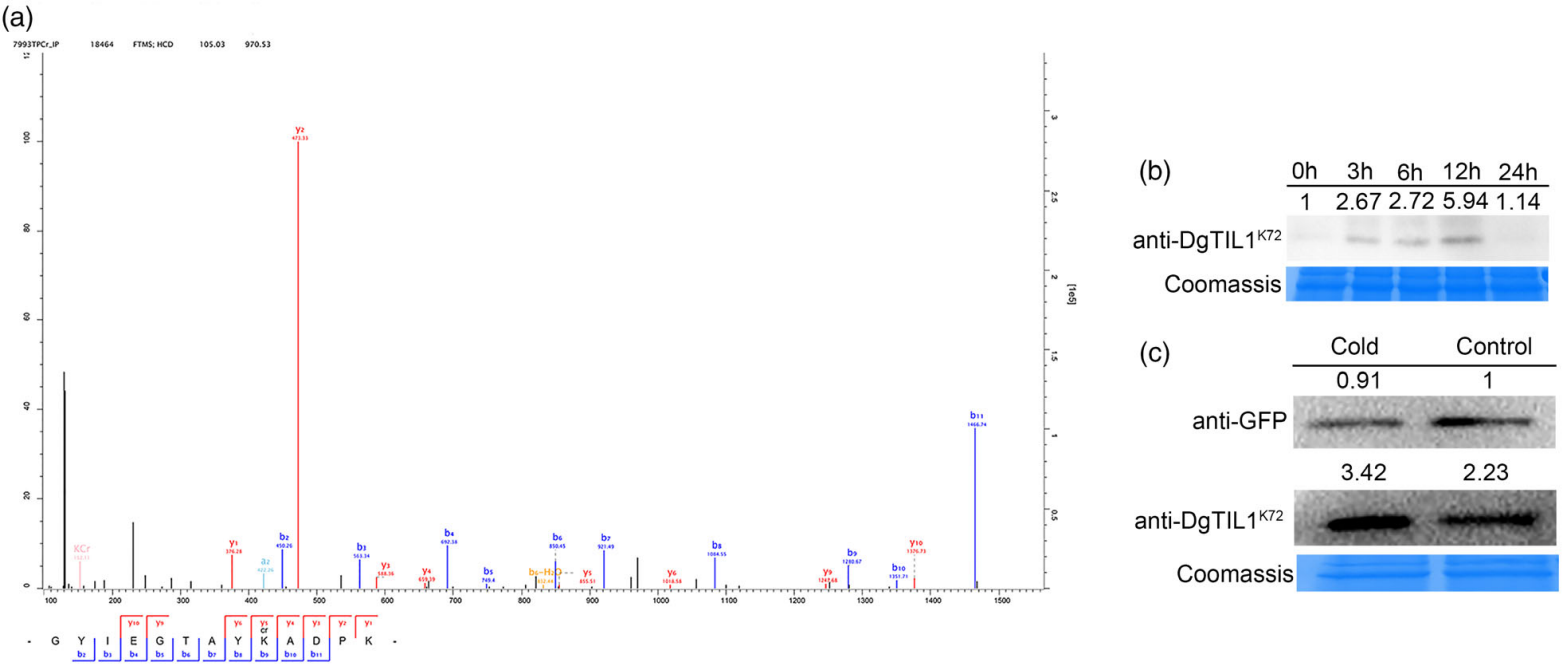
Cold induces lysine crotonylation of DgTIL1 at the K72 site (a) Dissociation mass spectrum revealed that the cold‐induced crotonylation site was the lysine (Lys) at residue 72 (K72) of the DgTIL1 protein that extracted from WT chrysanthemum with affinity purified. (b) Cold‐induced crotonylation of DgTIL1 protein from WT chrysanthemum increased with treatment time (hour) based on a western blot analysis of cold stress (4°C) using a specific anti‐DgTIL1^K72^ antibody (1:1000, from PTM BIO). (c) Immunoblot analysis of lysine crotonylation of the DgTIL1 protein at the K72 site in tobacco. *pSuper1300*‐DgTIL1‐GFP fusion protein was extracted from tobacco leaves and analysed with a GFP antibody (1:1000, from PTM BIO) and the specific antibody anti‐DgTIL1^K72^, and Coomassie blue staining was used to demonstrate consistent protein loading.

We performed lysine crotonylation‐modified TMT‐based quantitative proteome sequencing using cold‐treated (4°C for 24 h and −4°C for 4 h) and non‐treated (temperature, 25°C) chrysanthemum seedlings. We found that the K72 site of the DgTIL1 protein had a high aggregated crotonylated signal intensity (Figure 6a), and the signal intensity of the K72 site of the DgTIL1 protein was 0.76‐times higher than that of the control protein. The mass spectrometry proteomics data have been deposited in the ProteomeXchange consortium via the PRIDE partner repository under the dataset identifier PXD010297. Following crotonylation analysis, the specific antibody anti‐DgTIL1^K72^ was customized and used to label potential lysine‐crotonylated DgTIL1 proteins in the WT chrysanthemums (Figure 6b). Cold‐induced lysine crotonylation of the DgTIL1 protein in the WT chrysanthemums was successfully detected with the specific anti‐DgTIL1^K72^ antibody by Western blot analysis, and the level of crotonylation peaked at 12 h.

In addition, to verify whether lysine crotonylation of the DgTIL1 protein in tobacco leaves was induced under cold stress, the transient expression vector *pSuper1300‐DgTIL1‐GFP* was constructed to establish transient expression in tobacco leaves. Western blot experiments with anti‐GFP antibody revealed that compared with control conditions, low temperature had almost no effect on the level of the DgTIL1 protein in tobacco leaves. Based on the consistent protein level of DgTIL1, we found that the lysine crotonylation level of DgTIL1 was enhanced using the specific anti‐DgTIL1^K72^ antibody (Figure 6c), indicating that the DgTIL1 protein also undergoes lysine crotonylation under low temperature conditions in tobacco.

### Lysine crotonylation of DgTIL1 enhances the interaction between DgTIL1 and DgnsLTP and stabilizes DgnsLTP

**Figure 7:**
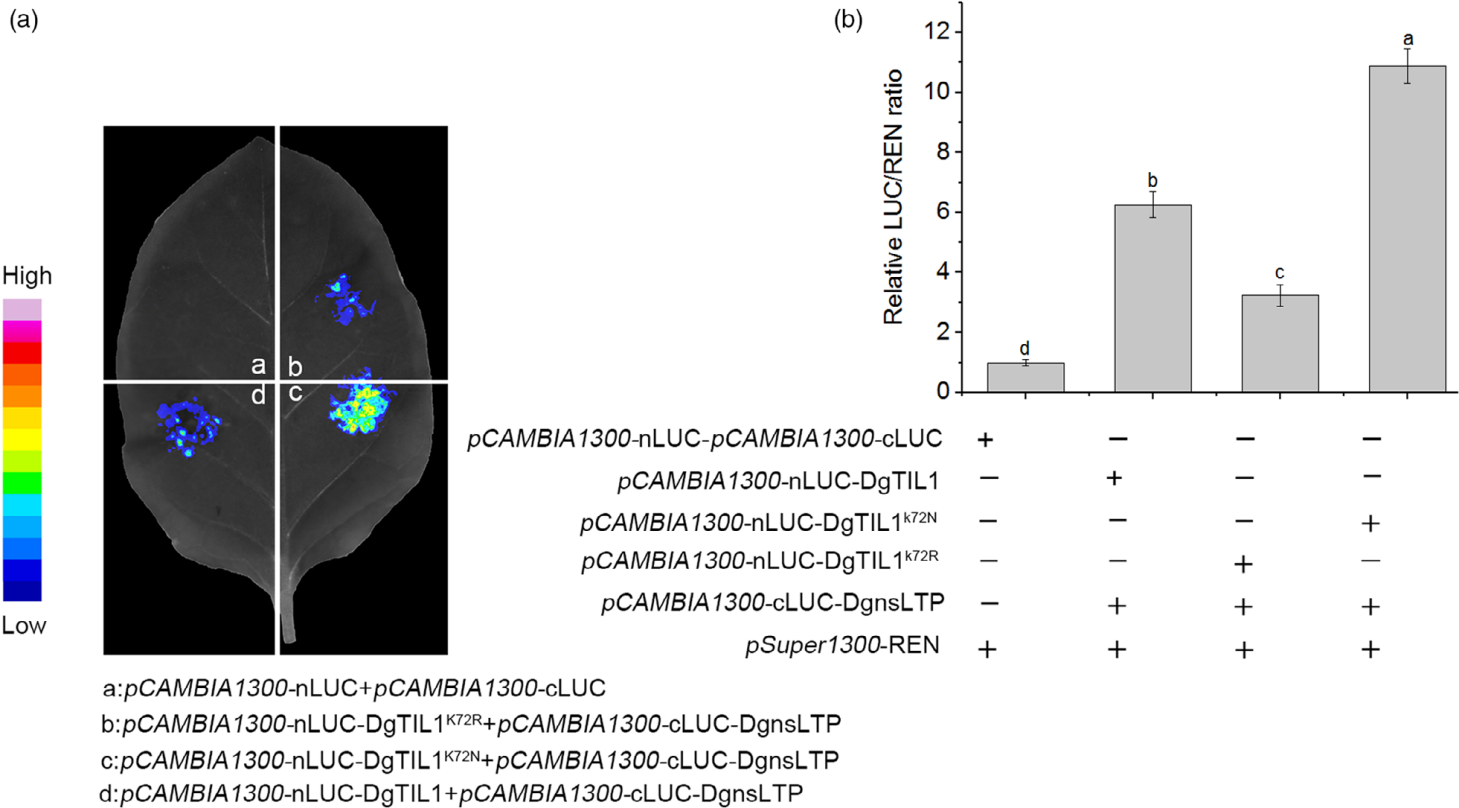
Lysine crotonylation at K72 strengthens the interaction of DgTIL1 with DgnsLTP (a) LCI assay shows the strength of the interaction between DgTIL1 and DgnsLTP. The N‐terminal (nLUC) or C‐terminal (cLUC) fragments of LUC were fused respectively with the indicated protein to transient coexpress. (b) DLA assay showing the interaction strength of relative reporter activity (LUC/REN) of the indicated fusion protein in tobacco, and the value of the empty vector was set to 1.

To evaluate whether the degree of crotonylation affects the interaction of DgTIL1 with DgnsLTP, we constructed crotonylation‐deficient and completely crotonylated forms of DgTIL1 by introducing mutations at the Lys (K) 72 site to either Arg (R) or Asn (N) (Figure S3a), which were named DgTIL1^K72R^ and DgTIL1^K72N^, respectively. We coexpressed *pCAMBIA1300*‐*DgTIL1*‐*nLUC*, *pCAMBIA1300*‐*DgTIL1^K72N^*‐*nLUC* or *pCAMBIA1300*‐*DgTIL1^K72R^*‐*nLUC* with *pCAMBIA1300*‐*DgnsLTP*‐*cLUC* and performed DLA and LCI assays in tobacco leaves. The *REN* reporter gene was coexpressed as an internal reference. The LCI assay results (Figure 7a) showed that the relative fluorescence of coexpressed *pCAMBIA1300*‐*DgTIL1*‐*nLUC* and *pCAMBIA1300*‐*DgnsLTP*‐*cLUC* was higher than that of coexpressed *pCAMBIA1300*‐*DgTIL1^K72R^*‐*nLUC* and *pCAMBIA1300*‐*DgnsLTP*‐*cLUC* but lower than that of coexpressed *pCAMBIA1300*‐*DgTIL1^K72N^*‐*nLUC* and *pCAMBIA1300*‐*DgnsLTP*‐*cLUC*. Additionally, the DLA experiments revealed similar trends (Figure 7b), indicating that the DgTIL1^K72N^ most strongly interacted with DgnsLTP and that the degree of crotonylation affected the interaction of DgTIL1 with DgnsLTP.

**Figure 8:**
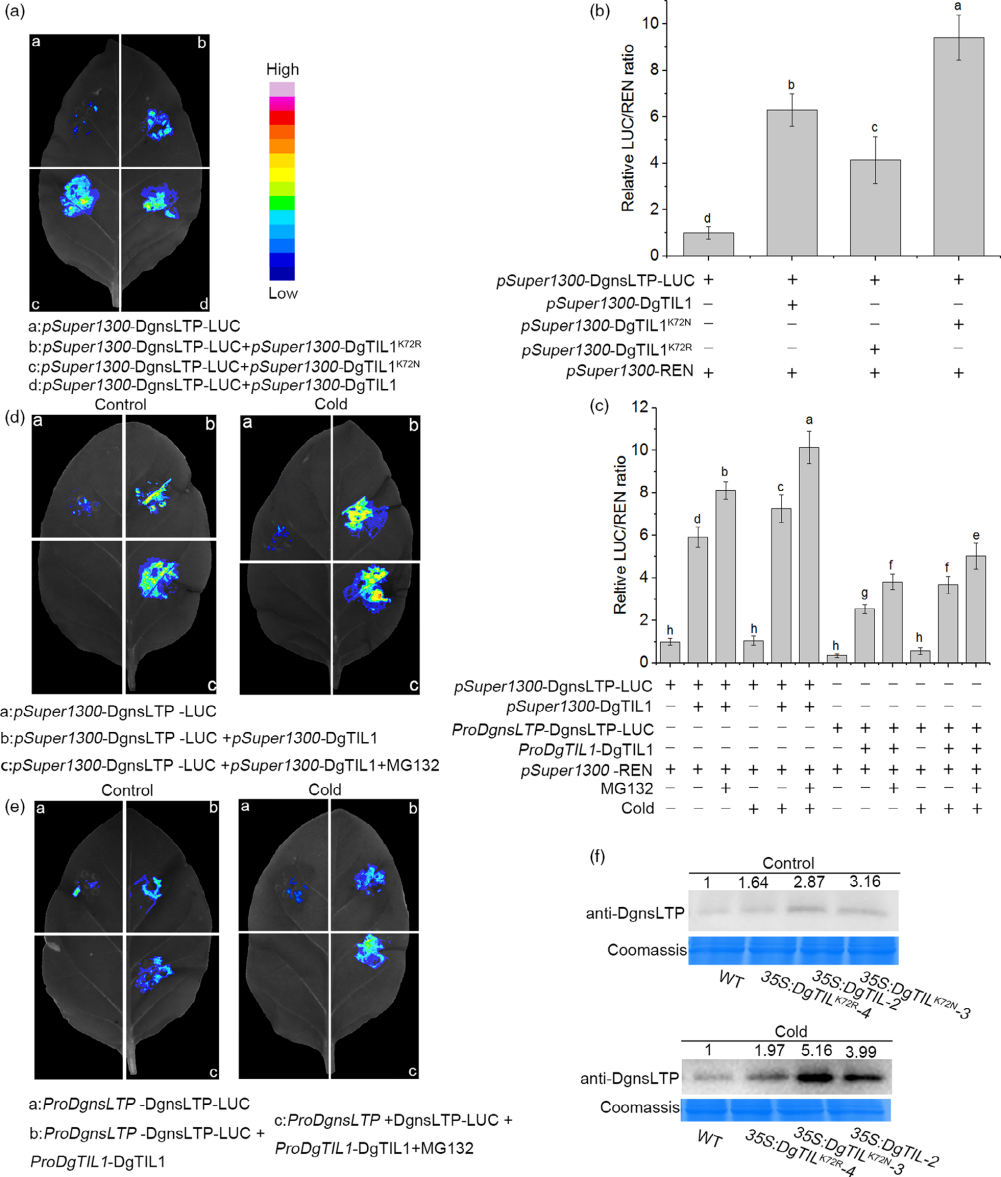
Cold‐induced lysine crotonylation of the DgTIL1 stabilizes DgnsLTP. (a and b) Activity of *pSuper1300*‐DgnsLTP‐LUC coexpressed with *pSuper1300*‐DgTIL1*,pSuper1300*‐DgTIL1^K72N^ *and pSuper1300‐*DgTIL1^K72R^ in tobacco was detected by LCI assay (a) and DLA assay (b); the fluorescent signal intensity indicates the activity of DgnsLTP under DgTIL1 inhibition, and the value of the empty vector was set to 1. (c, d and e) Comparison of the LUC activity of DgnsLTP‐LUC transiently coexpressed with *DgTIL1* between 35S promoter (*pSuper1300*) and the natural promoter under normal and low temperature treatment in tobacco. MG132 (20 μm) was used as one of the controls, (c) DLA assay, (d) driven by a 35S promoter, (e) driven by a natural promoter. (f) Expression of DgnsLTP protein in the WT and *DgnsLTP* transgenic chrysanthemum using anti‐DgnsLTP antibody (1:1000, from Sanon Biotech) under control conditions (25°C day/22°C night) and cold (4°C for 12 h) treatment, and Coomassie blue staining was used to demonstrate consistent protein loading.

To investigate whether the stability of DgnsLTP was affected by lysine crotonylation of DgTIL1, *pSuper1300*‐*DgTIL1*, *pSuper1300*‐*DgTIL1^K72N^* or *pSuper1300*‐*DgTIL1^K72R^* was coinjected with *pSuper1300*‐*DgnsLTP*‐*LUC* for a transient coexpression assay in tobacco leaves. *LUC* is a reporter gene, and whether the activity of *pSuper1300*‐DgnsLTP‐LUC was affected by different degrees of DgTIL1 modification was evaluated by LCI (Figure 8a). The highest fluorescence activity of *pSuper1300*‐DgnsLTP‐LUC was observed with DgTIL1^K72N^, followed by DgTIL1, and the lowest fluorescent activity was observed with DgTIL1^K72R^, which was consistent with the DLA results (Figure 8b). In summary, crotonylation of DgTIL1 was confirmed to affect the stability of DgnsLTP.

To further explore the function of lysine crotonylation of DgTIL1 at low temperatures, *pSuper1300*‐*DgTIL1* and *pSuper1300*‐*DgnsLTP*‐*LUC* were coexpressed in tobacco leaves. The relative fluorescence of coexpressed *pSuper1300*‐*DgnsLTP*‐*LUC* and *pSuper1300*‐*DgTIL1* was found to be significantly higher at low temperature than at control conditions (Figure 8c), indicating that lysine crotonylation of DgTIL1 at low temperature further inhibited DgnsLTP degradation. When MG132 was added, the inhibition of DgnsLTP protein degradation was more obvious. Under activation of the natural promoters of *DgTIL1* and *DgnsLTP*, DgTIL1‐mediated lysine crotonylation also prevented the degradation of DgnsLTP, although the relative fluorescence activity of coexpressed *ProDgnsLTP*‐*DgnsLTP*‐*LUC* and *ProDgTIL1*‐*DgTIL1* was lower than that of the 35S promoter. These results implied that DgnsLTP was possibly degraded via the 26S proteasome and that DgTIL1 lysine crotonylation at K72 inhibited this process. The LCI experiment showed the same result in a more intuitive manner (Figure 8d,e).

To further validate our experimental results, an anti‐DgnsLTP antibody was used for Western blotting to measure the protein level of DgnsLTP in *DgTIL1*, *DgTIL1^K72N^* and *DgTIL1^K72R^* transgenic chrysanthemum treated with cold stress (Figure 8f; Figure S3b). A comparison of the results under the control and low temperature conditions revealed that the *DgTIL1* transgenic lines expressed substantially more DgnsLTP *in vivo* than the *DgTIL1^K72R^* transgenic lines and the WT lines under low temperature but expressed less DgnsLTP than the *DgTIL1^K72N^* transgenic lines, indicating that the degree of lysine crotonylation of DgTIL1 can affect the protein expression of DgnsLTP in chrysanthemum.

### Inhibition of degradation of DgnsLTP by lysine crotonylation of DgTIL1 further enhances POD activity and minimizes ROS in *DgTIL1* transgenic chrysanthemum

**Figure 9:**
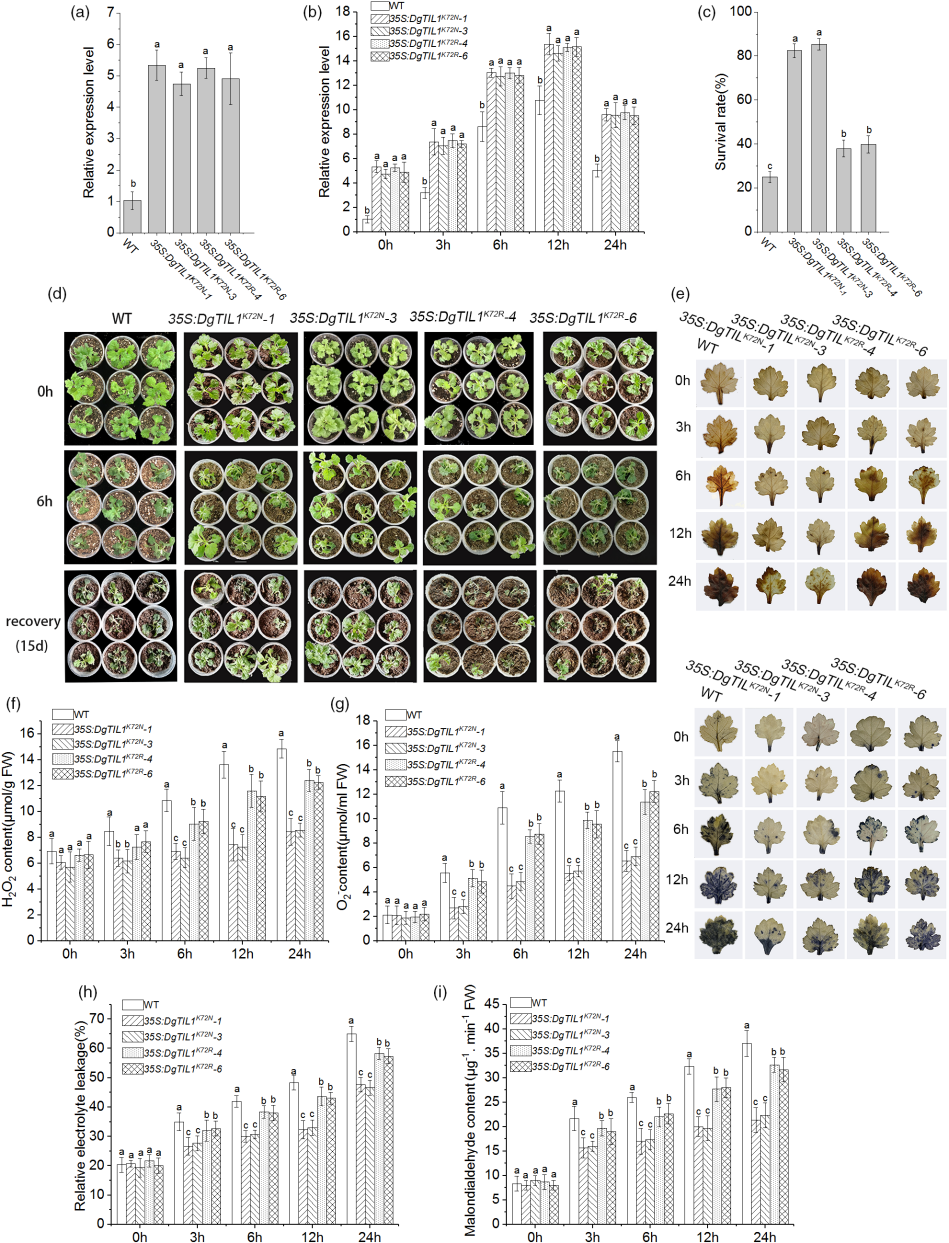
Lysine crotonylation of DgTIL1 at K72 enhances the cold resistance in chrysanthemum. (a and b) Expression levels of *DgTIL1* in the WT and transgenic chrysanthemum under control conditions (25°C day/22°C night) (a) and low temperature stress at 4°C (b) using qRT‐PCR (*P *< 0.05) (3 replicates, 20 plants per replicate). (c) Survival rates of chrysanthemum after 15 days of recovery under control conditions (25°C day/22°C night). (d) Chrysanthemum phenotype changes under low temperature stress at −6°C for 8e and recovery. (e) Histochemical staining was used to reveal the accumulation of H_2_O_2_ and O_2_^‐^ with DAB and NBT in the WT and transgenic lines. (f and g) Analysis of the accumulation of ROS in the WT and transgenic lines as detected by quantitative measurement, (f) content of H_2_O_2_, (g) content of O_2_^‐^ (3 replicates, 50 plants per replicate). (h) Relative electrolyte leakage. (i) Malondialdehyde content (3 replicates, 50 plants per replicate).

To further investigate how lysine crotonylation at K72 affects the function of DgTIL1 in cold tolerance, *35S::DgTIL1^K72R^* and *35S::DgTIL1^K72N^* were constructed and transformed into WT chrysanthemum. The *35S::DgTIL1^K72N^* transgenic lines (*35S::DgTIL1^K72N^‐1* and *35S::DgTIL1^K72N^‐3*), *35S::DgTIL1^K72R^* transgenic lines (*35S::DgTIL1^K72R^‐4* and *35S::DgTIL1^K72R^‐6*) and *35S::DgTIL1* transgenic lines (*35S::DgTIL1‐2* and *35S::DgTIL1‐4*) were treated with low temperature. The qRT‐PCR revealed that the relative expression of *DgTIL1* in the *DgTIL1^K72R^* transgenic lines and *DgTIL1^K72N^* transgenic lines was significantly (*P *< 0.05) higher than that in the WT lines under cold treatment, although distinct differences were not observed among the *35S::DgTIL1^K72N^*, *35S::DgTIL1^K72R^* and *35S::DgTIL1* transgenic lines (Figure 9a,b; Figure 2a). Furthermore, the *DgTIL1* transgenic lines had higher survival than the *DgTIL1^K72R^* transgenic and WT lines but lower survival than the *DgTIL1^K72N^* transgenic lines (Figure 9c,d; Figure 2b). Histochemical staining and H_2_O_2_ and O_2_^‐^ content measurements both revealed that all transgenic lines and the WT lines showed an increasing trend with increasing cold treatment time (Figure 9e–g); moreover, the *DgTIL1* transgenic lines accumulated less H_2_O_2_ and O_2_^‐^ than the *DgTIL1^K72R^* transgenic lines and the WT lines but more H_2_O_2_ and O_2_^‐^ than the *DgTIL1^K72N^* transgenic lines. The malondialdehyde and relative electrolyte leakage trends were similar to that of the H_2_O_2_ and O_2_^‐^ results, with a lower relative electrolyte leakage level and less malondialdehyde accumulation observed in the *DgTIL1^K72N^* transgenic lines than the *DgTIL1* and *DgTIL1^K72R^* transgenic lines (Figure 9h–i; Figure 2g–h).

**Figure 10:**
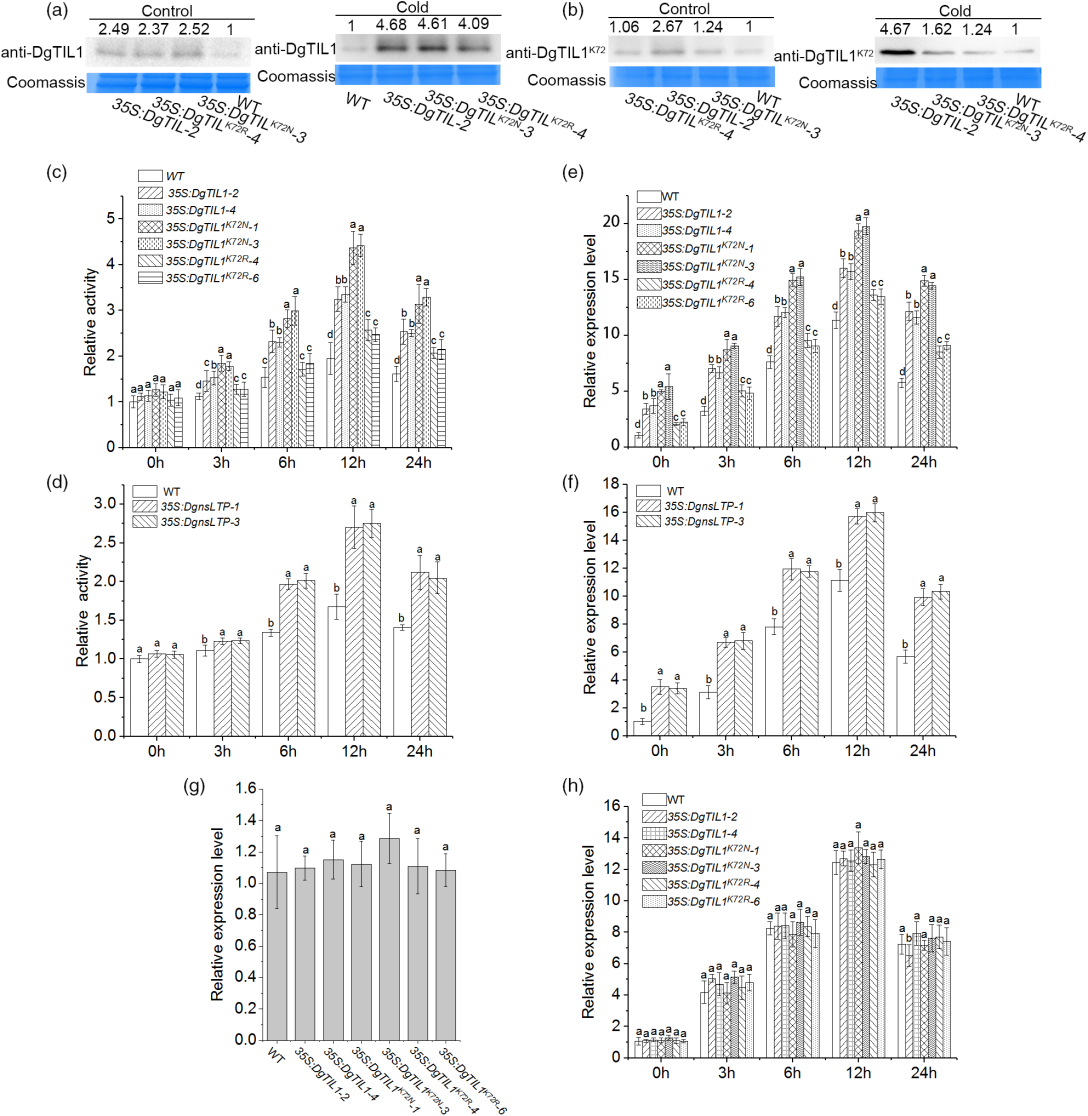
Inhibition of degradation of DgnsLTP by lysine crotonylation of DgTIL1 further enhanced the POD activities (a) Protein abundance of WT and transgenic chrysanthemum as detected by anti‐DgTIL1 antibody under the control and low temperature conditions at 4°C for 12 h. (b) Immunoblot analysis of lysine crotonylation of the DgTIL1 protein in transgenic chrysanthemum and WT chrysanthemum using the anti‐DgTIL1^K72^ antibody under the control and low temperature conditions at 4°C for 12 h. (c and d) Activity of POD in the WT chrysanthemum, *DgTIL1, DgTIL1^K72N^, DgTIL1^K72R^* transgenic chrysanthemum (c) and *DgnsLTP* transgenic chrysanthemum (d) under cold stress (4°C), with 3 replicates and 50 plants per replicate. (e and f) Expression level of *POD* in the WT chrysanthemum, *DgTIL1, DgTIL1^K72N^, DgTIL1^K72R^* transgenic chrysanthemum (e) and *DgnsLTP* transgenic chrysanthemum (f) under cold stress (4°C), with 3 replicates and 20 plants per replicate. (g and h) *DgnsLTP* expression level in the WT and transgenic chrysanthemum under control conditions (25°C day/22°C night) (g) and induced by low temperature stress (4°C) (h) (*P *< 0.05), with 3 replicates and 20 plants per replicate.

At the protein level, immune experiments using anti‐DgTIL1 antibody revealed that the three transgenic chrysanthemum lines (*35S::DgTIL1*, *35S::DgTIL1^K72N^* and *35S::DgTIL1^K72R^*) produced more DgTIL1 protein than the WT lines under the control and low temperature conditions (Figure 10a). Additionally, the abundance of the DgTIL1 protein was almost consistent in these transgenic chrysanthemum (Figure 10a; Figure S4a). Therefore, the cold tolerance differences in the *DgTIL1, DgTIL1^K72N^* and *DgTIL1^K72R^* transgenic chrysanthemums lines are related to the differences in DgnsLTP protein degradation *in vivo* (Figure 8f).

At the modification level, the proteins extracted from the three transgenic chrysanthemums were subjected to immunoblot experiments with the anti‐DgTIL1^K72^ antibody (Figure 10b; Figure S4b). The results showed that the anti‐DgTIL1^K72^ antibody successfully detected the lysine crotonylation of the DgTIL1 protein under the control and low temperature conditions, although no significant difference was identified among the *DgTIL1^K72R^* and *DgTIL1^K72N^* transgenic lines and the WT lines.

To explore the mechanism of ROS‐scavenging enzymes, we measured the activity of POD, APX and CAT enzymes in the overexpression and WT lines under the low temperature and control conditions. The results showed that the activity of POD was highest in the *DgTIL1^K72N^* transgenic lines that showed less degradation of DgnsLTP protein. Lower DgnsLTP protein degradation was found in the *DgTIL1* transgenic lines than the *DgTIL1^K72R^* transgenic lines; therefore, the activity of POD in the *DgTIL1* transgenic lines was higher than that of the *DgTIL1^K72R^* transgenic lines (Figure 10c). Furthermore, the *DgnsLTP* transgenic lines also showed increased POD activity than the WT lines (Figure 10d), which reduced the ROS toxicity. However, the activity of APX and CAT enzymes in the *DgTIL1*, *DgTIL1^K72N^*, *DgTIL1^K72R^*, *DgnsLTP* transgenic lines and WT lines did not change significantly (Figure S5a‐d). In addition, the expression of *POD* was consistent with the trend of POD activity (Figure 10e,f), thus indicating that DgnsLTP increases the expression of *POD* to enhance the cold resistance of the *35S::DgTIL1*, *35S::DgTIL1^K72N^*, *35S::DgTIL1^K72R^* and *35S:: DgnsLTP* transgenic lines.

A qRT‐PCR assay was performed to verify the effect of the DgTIL1 on *DgnsLTP* expression in the *DgTIL1*, *DgTIL1^K72N^* and *DgTIL1^K72R^* transgenic lines, and almost no differences (*P *< 0.05) in the expression of *DgnsLTP* was observed between the transgenic lines and the WT line under the control conditions (Figure 10g) and low temperature conditions (Figure 10h), suggesting that DgTIL1 did not affect the gene expression of *DgnsLTP* in these transgenic lines.

Overall, the lysine crotonylation modification of DgTIL1 enhanced the ability of DgnsLTP to remove ROS in chrysanthemum and improved the cold resistance of chrysanthemum, with a higher modification level corresponding to less DgnsLTP degradation and stronger cold resistance.

## Discussion

To date, *TILs* have been studied in a variety of plants, including *Arabidopsis,* wheat (Abo‐Ogiala *et al*., 2014; Boca *et al*., 2013; Charron *et al*., 2002; Chi *et al*., 2009), *M. falcate* (He *et al*., 2005), *Narcissus tazetta* (Ding *et al*., 2016) and other plants, and these genes respond to a variety of abiotic stresses. We isolated a temperature‐induced lipocalin‐1‐like gene named *DgTIL1* from chrysanthemum. A sequence analysis and phylogenetic analysis revealed that this protein contains three conserved SCR domains that are characteristic of lipoproteins (Flower *et al*., 2000) and closely homologous to plant temperature‐induced lipocalin‐1‐like proteins (Figure S1a,b). TILs are plasma membrane proteins that can increase the cryostability of the plasma membrane during low temperature treatment (Uemura *et al*., 2006). *TaTIL1* can respond to temperature stress in wheat, and the transcript accumulation of *TaTIL1* was nearly 10‐times higher after low temperature stress compared with the control conditions (Charron *et al*., 2002; Charron, 2005). Furthermore, *Arabidopsis AtTIL1* has high homology with wheat *TaTIL1* (Chi *et al*., 2009; Charron, 2005), and the role of *AtTIL1* in freezing stress tolerance using *AtTIL1* transgenic *Arabidopsis* has been reported (Charron *et al*., 2008). *AtTIL1* can participate in low temperature stress processes by promoting transcription (Charron *et al*., 2002), and the protein level of AtTIL1 is also significantly induced by low temperature (Kawamura and Uemura, 2003). In contrast, knockout mutants of *AtTIL1* in *Arabidopsis* under low temperature stress accumulated more peroxidation products and were more sensitive to cold stress than the WT line (Charron *et al*., 2008; Chi *et al*., 2009). In addition, overexpression of *MfTIL1* can also improve the cold tolerance of plants (He *et al*., 2005). However, the function of the *DgTIL1* gene in response to environmental stress in chrysanthemum remains poorly understood.

In our research, the transcription level of *DgTIL1* was up‐regulated and the protein level of DgTIL1 was induced by low temperature in the WT chrysanthemum (Figure 1b,c and f). The *DgTIL1* transgenic lines were compared with the WT lines at the transcriptome, protein and physiological levels, and the cold resistance of *DgTIL1* transgenic lines was evaluated. After low temperature treatment, the relative expression level of *DgTIL1* in transgenic chrysanthemum was significantly up‐regulated compared with that in the WT lines (Figure 2a), and the protein abundance of DgTIL1 in the transgenic lines was also higher than that in the WT lines (Figure 10a; Figure S4a). Moreover, both the relative electrolyte leakage and content of malondialdehyde continued to increase under cold stress in the WT and transgenic chrysanthemum leaves (Figure 2g,h). We speculated that cold stress causes marked accumulation of ROS (such as H_2_O_2_ and O_2_^‐^) in chrysanthemum because high ROS accumulation causes toxicity to plant cells. Electrolyte leakage and malondialdehyde are used as indicators of ROS accumulation levels and can reflect changes in membrane permeability and lipid peroxidation (Farmer and Mueller, 2013; Mittler *et al*., 2004). The results showed that ROS accumulation increases in chrysanthemum under cold stress (Figure 2d‐f); however, lower relative electrolyte leakage, malondialdehyde contents (Figure 2g,h) and ROS (such as H_2_O_2_ and O_2_^‐^) accumulation (Figure 2d–f) were observed in the *DgTIL1* transgenic lines relative to the WT lines, which is consistent with previous reports indicating that *TILs* can enhance the stability of cell membranes at low temperatures (Chi *et al*., 2009; Kawamura and Uemura, 2003; Uemura *et al*., 2006). Maintaining the ROS balance in cells is the key to plant survival. In this process, antioxidants and antioxidative enzymes play a vital role in removing accumulated ROS. POD is a key antioxidant enzyme that eliminates ROS (Gao *et al*., 2010; Passardi *et al*., 2004). In our study, the activity of POD was increased in the *DgTIL1* transgenic lines compared with the WT lines and the relative expression level of *POD* was up‐regulated (Figure 10c,e). These changes eliminated ROS toxicity and improved the cold resistance in chrysanthemum.

To better explore the regulated mechanism of DgTIL1 in response to cold stress, we identified a nonspecific nsLTP that interacts with DgTIL1 using the MYTH system (Table S2). nsLTPs are a small family of basic proteins (Stergaard *et al*., 1993) that can increase the cold resistance of plants. For instance, overexpressing *OsLTPL159* in rice can improve cold tolerance (Zhao *et al*., 2020). In *A. thaliana*, the promoter of *lipid transfer protein 3* (*LTP3)* is regulated by MYB96 and overexpression of *LTP3* can improve the cold tolerance of plants (Guo *et al*., 2013). In our study, we isolated an *nsLTP* gene from chrysanthemum, *DgnsLTP*, which was dramatically induced by low temperature and up‐regulated rapidly in the gene expression level the and in the protein level in the WT chrysanthemum (Figure 4b–c and f). Furthermore, the *DgnsLTP*‐overexpressing chrysanthemums showed enhanced tolerance to low temperature stress (Figure 5a‐c). In addition, overexpression of *DgnsLTP* can reduce the accumulation of ROS (H_2_O_2_, O_2_^‐^, relative electrolyte leakage, malondialdehyde) by regulating POD activity and enhancing *POD* expression (Figure 5d–h; Figure 10d,f). Therefore, *DgnsLTP* may be an important regulator of cold stress.

In our research, we further evaluated the interactions of DgnsLTP with DgTIL1 on the plasma membrane through Y2H experiments and a BIFC analysis (Figure 3a,b). In addition, we investigated whether the stability of DgnsLTP was influenced by DgTIL1 in tobacco and chrysanthemum. We found that when coexpressing *pSuper1300*‐*DgTIL1* and *pSuper1300*‐*DgnsLTP*‐*LUC* in tobacco, the LUC activity of *pSuper1300*‐DgnsLTP‐LUC was higher than that without coexpression of *pSuper1300*‐*DgTIL1*; moreover, the LUC activity of *ProDgnsLTP*‐DgnsLTP‐LUC, which is driven by the natural promoter of *DgnsLTP*, was not as obvious as the results driven by the super promoter. However, the activity was also affected by *ProDgTIL1‐DgTIL1* (Figure 8c–e), indicating that DgTIL1 not only interacts with DgnsLTP but also affects the protein stability of DgnsLTP in tobacco. Furthermore, the abundance of the DgnsLTP protein in *DgTIL1* transgenic chrysanthemum was higher than that in the WT lines, and more protein accumulated after low temperature induction (Figure 8f; Figure S3b). Therefore, we concluded that DgTIL1 can affect the protein stability of DgnsLTP in chrysanthemum. In addition, we found that the transcript level of *DgnsLTP* in *DgTIL1* transgenic chrysanthemum was not significantly different from that in the WT lines (Figure 10g,h); therefore, we determined that DgTIL1 only affected the protein level of DgnsLTP. Regarding the cold‐stress regulation mechanism, we found that the expression level of *POD* and activity of POD in the *DgTIL1* transgenic lines and *DgnsLTP* transgenic lines were both induced by low temperature (Figure 10c–f), indicating that the cold‐stress regulation mechanism of these lines is regulated by the *POD* gene and POD activity. Therefore, we speculated that the enhanced cold resistance of the *DgTIL1* transgenic lines was associated with enhanced POD activity and up‐regulated *POD* expression, which are related to the protein stability of DgnsLTP.

Increasing evidence suggests that PTMs of nonhistone proteins can be involved in abiotic stress processes; for example, ubiquitination and phosphorylation have been widely reported to be involved in low temperature stress (Sun *et al*., 2017; Tan *et al*., 2011). These modifications changed the localization and activity status of the modified protein and can also regulate the function of the protein through the protein interaction network (Liu *et al*., 2018a; Lu *et al*., 2018; Xu *et al*., 2017). Evolutionarily conserved lysine crotonylation is a newly discovered type of PTM (Huang *et al*., 2018). Histone and nonhistone protein lysine crotonylation has been reported from yeast to plants to be involved in transcriptional regulation, photosynthesis, carbon fixation, amino acid metabolism and other biological processes (Liu *et al*., 2018a; Lu *et al*., 2018; Tan *et al*., 2011). In addition, a protein interaction network analysis revealed that nonhistone protein crotonylation can affect not only protein interactions but also enzyme activity through these biological processes (Xu *et al*., 2017). However, whether nonhistone proteins with lysine crotonylation play a role in cold stress has not been reported. In our research, we found that lysine crotonylation of *DgTIL1* occurred at the K72 site under low temperature induction (Figure 6a–c). Additionally, the *DgTIL1*, *DgTIL1^K72N^* and *DgTIL1^K72R^* transgenic lines have different cold tolerances and the *DgTIL1^K72N^* transgenic lines have a higher survival rate than the *DgTIL1* and *DgTIL1^K72R^* transgenic lines (Figure 2b; Figure 9c). Moreover, in the *DgTIL1^K72N^* transgenic lines, the relative electrolyte leakage, malondialdehyde contents and ROS accumulation (H_2_O_2_ and O_2_^‐^) were significantly lower than those in the *DgTIL1* and *DgTIL1^K72R^* transgenic lines (Figure 2d–h; Figure 9e–i) and the POD activity and *POD* expression level were higher than those in the *DgTIL1* and *DgTIL1^K72R^* transgenic lines (Figure 10c,e). Therefore, the *DgTIL1^K72N^* transgenic lines had the highest cold tolerance. However, ROS accumulation (H_2_O_2_ and O_2_^‐^ contents, relative electrolyte leakage, the malondialdehyde content) in the *DgTIL1^K72R^* transgenic lines were higher than those in the *DgTIL1* and *DgTIL1^K72N^* transgenic lines (Figure 2d–h; Figure 9e–i), and POD activity and *POD* expression in *DgTIL1^K72R^* transgenic lines were lower than those in the *DgTIL1* and *DgTIL1^K72N^* transgenic lines (Figure 10c,e). Therefore, the *DgTIL1^K72R^* transgenic lines have lower cold tolerance than the *DgTIL1* and *DgTIL1^K72N^* transgenic chrysanthemum lines. However, slightly stronger resistance to cold stress is observed in *DgTIL1^K72R^* transgenic lines compared with the WT lines, and we speculate that DgTIL1‐mediated cold resistance is only partially mediated by the crotonylation of K72 site. The expression level of *DgTIL1* among the *DgTIL1, DgTIL1^K72R^* and *DgTIL1^K72N^* transgenic lines was nearly consistent (Figure 2a; Figure 9b), and the protein level of DgTIL1 was almost the same (Figure 10a; Figure S4a). Moreover, significant differences in the transcription level of *DgnsLTP* were not observed between the *DgTIL1*, *DgTIL1^K72N^* and *DgTIL1^K72R^* transgenic lines and the WT lines (Figure 10g,h). Therefore, we further clarified that the different cold tolerances are related to lysine crotonylation of DgTIL1. Further experiments showed different interaction strengths between DgTIL1 and DgnsLTP with different degrees of lysine crotonylation of DgTIL1. Coexpressing *pCAMBIA1300*‐*DgTIL1^K72N^*‐*nLUC* with *pCAMBIA1300*‐*DgnsLTP*‐*cLUC* yielded the strongest interaction, thus proving that a stronger degree of lysine crotonylation of DgTIL1 corresponds to a stronger interaction with DgnsLTP (Figure 7a,b). Cold stress can induce lysine crotonylation of DgTIL1 in tobacco, preventing the degradation of DgnsLTP (Figure 6c; Figure 8c–e). Moreover, among the coinjection treatments, LUC activity was the highest when *pSuper1300*‐DgTIL1^K72N^ was coinjected with *pSuper1300*‐DgnsLTP‐LUC in tobacco (Figure 8a,b), and the inhibition of DgnsLTP protein degradation was more obvious in *DgTIL1^K72N^* transgenic chrysanthemum than in the other lines (Figure 8f). The activity of *pSuper1300*‐DgnsLTP‐LUC regulated by *pSuper1300*‐DgTIL1^K72R^ in tobacco, and the abundance of the DgnsLTP protein in *DgTIL1^K72R^* transgenic chrysanthemum presented opposite results compared with that observed in coexpressing *pSuper1300*‐DgTIL1^K72N^ with *pSuper1300*‐DgnsLTP‐LUC in tobacco and in *DgTIL1^K72N^* transgenic chrysanthemum (Figure 8a,b and f). This result indicated that a higher degree of crotonylation of DgTIL1 corresponds to lower degradation of the DgnsLTP protein. DgnsLTP further promotes the expression of *POD* and removes accumulated ROS in chrysanthemum under low temperature stress, thus improving the cold resistance of chrysanthemum. Overall, the above results clarified that a higher degree of crotonylation of DgTIL1 corresponds to stronger protein stability of DgnsLTP, which induced higher POD activity and *POD* transcription associated with DgnsLTP, thereby increasing the cold resistance of chrysanthemum. Although the pathway of DgnsLTP‐induced *POD* expression is not clear and requires subsequent verification, these results reveal a new method used by chrysanthemum to resist low temperature stress and provide new insights for the molecular breeding of chrysanthemum.

**Figure 11:**
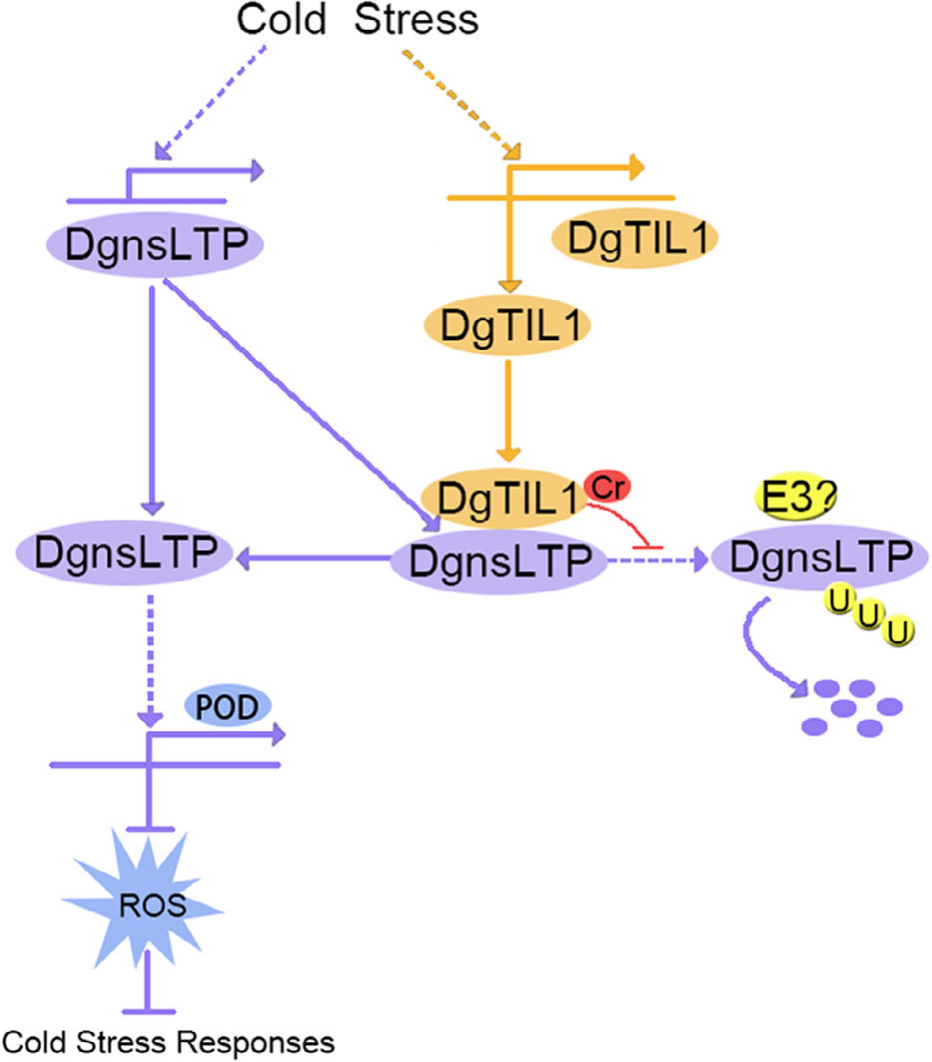
Proposed model for DgTIL1‐mediated DgnsLTP modulation of cold stress. Cold stress induces crotonylation of DgTIL1 at the Lys 72 site, which enhances the interaction between DgTIL1 and DgnsLTP and prevents the degradation of the interacting protein DgnsLTP. These changes allow DgnsLTP to activate *POD* expression and POD activity and thus improve the cold resistance of chrysanthemum.

In our study, a cold stress‐responsive gene, *DgTIL1,* was identified. Overexpression of *DgTIL1* improved the cold resistance of chrysanthemum. The DgnsLTP protein interacts with DgTIL1 and can actively respond to low temperature. Lysine crotonylation of DgTIL1 at the K72 site can improve the stability of the DgnsLTP protein and affect the activity of POD and the expression of *POD* related to DgnsLTP, thus improving the cold resistance of chrysanthemum (Figure 11).

## Experimental procedures

### Plant material preparation and low temperature treatment

*Chrysanthemum morifolium* var. Jinba was the main plant material using in this study. The top buds of the chrysanthemums were cultured on MS medium for 20 days and then used as material for the genetic transformation experiments. Chrysanthemum seedlings with 7–8 leaves were transplanted into 1:1 mixed peat and perlite and cultivated in biochemical incubators for 3 days (23 ± 2°C, 75% relative humidity), and then low temperature treatments with different time gradients (4°C for 0 h, 3 h, 6 h, 12 h and 24 h, 75% relative humidity) were performed. Subsequently, the seedlings were subjected to total protein and RNA extraction and physiological index measurements. After the freezing treatment (−6°C for 6 h) phenotype changes were observed. The plants were then allowed to recover for 15 days under control conditions (25°C day/22°C night) to detect the survival rates.

### RNA extraction and quantitative real‐time polymerase chain reaction (qRT‐PCR) assay

The total RNA of chrysanthemum was extracted via a Spin Column Plant Total RNA Purification kit (Sangon Biotech, Shanghai, China) and prepared using a One‐Step gDNA Removal kit (Transgen Biotech, Beijing, China) based on the manufacturers’ instructions. Then, cDNA was added to the mixture according to the quantitative kit operation system (Transgen Biotech) to conduct qRT‐PCR with a Bio‐Rad CFX96™ detection system. The elongation factor 1α (*EF1α*) gene was selected as a stable reference gene, and the 2‐ΔΔCT method was used to analyse the results. The primers for gene amplification are shown in Table S3.

### Construction of the *DgTIL1* and *DgnsLTP* expression vectors and acquisition of transgenic chrysanthemum

To overexpress *DgTIL1*, *DgTIL1^k72N^*, *DgTIL1^K72R^* and *DgnsLTP* in chrysanthemum (variety ‘Jinba’), the full‐length cDNA was double‐digested with Sall/SpeI and inserted into the pSuper1300 vector, with the specific operation completed by Sanon Biotech. The obtained recombinant plasmids *pSuper1300‐DgTIL1*, *pSuper1300‐DgTIL1^k72N^*, *pSuper1300‐DgTIL1^K72R^* and *pSuper1300‐DgnsLTP* were transformed into chrysanthemum according to previously reported steps to obtain transgenic chrysanthemum (An *et al*., 1986).

### Transient expression assays

Transient expression was performed in the leaves of *N. benthamiana* at the 5‐6 leaf stage according to a previously reported method (An *et al*., 2017). The fusion protein was constructed and coinjected into tobacco leaves, and 3 days later (23 ± 2°C, 75% relative humidity), the protein was used for subsequent experiments.

### Protein extraction and Western blotting

Proteins from the low temperature treatments with different time gradients (4°C for 0 h, 3 h, 6 h, 12 h and 24 h) were extracted from the samples at each time gradient, which were stored at −80°C and then prepared according to the instructions of the plant total protein extraction kit (BestBio, Shanghai, China). The extracted proteins were measured by a microplate reader (Thermo Fisher Multiskan GO，Waltham, Massachusetts, USA) according to the instructions of the protein quantification kit (BCA) (Transgen Biotech, Beijing, China).

The anti‐DgTIL1 antibody and a specific anti‐DgTIL1^K72^ antibody were customized by PTM BIO Company (Hangzhou, China), the anti‐GFP antibody was purchased from Transgen Biotech Company, and the anti‐DgnsLTP antibody was customized by Sangon Biotech Company. DgTIL1 and DgnsLTP protein abundances were separately measured by Western blot analysis using the anti‐DgTIL1 antibody and anti‐DgnsLTP antibody. A 15% SDS‐PAGE gel was used for protein separation, 0.22‐µm PVDF membranes were used to transfer the membrane, and the transferred membrane was incubated with the aforementioned primary antibody (1:1000). After washing the membrane in TBST and secondary antibody (Immunoway, USA) incubation (1: 10000), images were finally developed on an imager with a Western blot kit (Transgen Biotech, Beijing, China).

### Subcellular localization

Full‐length *DgTIL1* and *DgnsLTP* were cloned into the transient expression vector *pSuper300*‐GFP at the Sacl/salI site. Then, *Agrobacterium tumefaciens* GV3101 was cotransformed with the recombinant plasmid, and the gene bacterial fluid and membrane localization marker (CD3‐1007) were cotransformed into tobacco leaves to execute transient expression (An *et al*., 2017). The results were observed by laser scanning confocal microscopy (LSCM) after 3 days.

### Y2H Assay

The Y2H test was conducted in line with previously reported methods (Nan *et al*., 2012). Full‐length *DgTIL1* was added to the *pBT3‐N* vector using the Sacl/salI digestion site, and full‐length *DgnsLTP* was added to the *pPR3‐N* vector using the EcoRI/SalI digestion site. *pBT3‐N‐DgTIL1* plasmids were cotransformed with *pPR3‐N‐DgnsLTP* into strain AH109. *pBT3‐N‐DgTIL1* + *pPR3‐N*, *pBT3‐N* + *pPR3‐N‐DgnsLTP* and *pTSU2*‐*APP *+ *pPR3‐N* were used as negative controls, and *PTSU2*‐*APP *+ *pNubG*‐*Fe65* was used as a positive control.

### BIFC

Using PCR‐based accurate synthesis (PAS) to design full‐length splicing primers, protective bases were designed on both ends of the primers to synthesize the *DgnsLTP* and *DgTIL1* genes. Correspondingly, the EcoRI‐SalI site of the vector *pCAMBIA1300*‐*35S*‐*YFPc* and the EcoRI‐SalI site of *pCAMBIA1300*‐*35S*‐*YFPn* were linked, and then the recombinant plasmids *pCAMBIA1300*‐*35S*‐*YFPc*‐*DgnsLTP* and *pCAMBIA1300*‐*35S*‐*YFPn*‐*DgTIL1* were obtained. The experiment was performed according to a previously reported method (An *et al*., 2018).

### Construction of expression vectors, luciferase complementation imaging and LUC/REN activity analysis

For construction of the experimental vector used to verify the strength of the interaction, full‐length *DgTIL1* and crotonylation‐deficient and lysine complete crotonylation sequences were added to *pCAMBIA1300*‐*nLUC* using the Sacl/salI restriction site, full‐length *DgnsLTP* was added to *pCAMBIA1300*‐*cLUC* using the KpnI/SalI restriction site, and the internal reference gene REN was added to *pSuper1300* using SalI/KpnI digestion sites, which resulted in the recombinant plasmids *pCAMBIA1300*‐*nLUC*‐*DgTIL1* and *pCAMBIA1300*‐*cLUC*‐*DgnsLTP*.

To construct the experimental vectors used to measure DgnsLTP‐LUC activity, the full‐length *DgnsLTP* gene and *LUC* were added to the same vector, namely *pSuper1300*, using the SalI/SpeI restriction site and the SalI/KpnI restriction site, respectively. *DgTIL1* was added to *pSuper1300* using the SalI/KpnI restriction site, which resulted in the recombinant plasmids *pSuper1300*‐*DgnsLTP*‐*LUC* and *pSuper1300*‐ *DgTIL1*. The *REN* reporter gene was coexpressed as an internal reference. To construct a vector with a natural promoter, the natural promoters of *DgTIL1* and *DgnsLTP* (1.5 kb) were cut into the *pSuper1300* vector using the BamH1/Kpn1 restriction site. *LUC* was added to the same vector, *pSuper1300*, using the SalI/SpeI restriction site, resulting in the recombinant plasmids *ProDgTIL1*‐LUC and *ProDgnsLTP*‐*LUC*. *DgTIL1* was used to construct *ProDgTIL1*‐*LUC* with the SalI/KpnI restriction site, and the recombinant plasmid *ProDgTIL1*‐*DgTIL1*‐*LUC* was obtained. *DgnsLTP* was used to construct *ProDgnsLTP*‐*LUC* by the SalI/SpeI restriction site, and the recombinant plasmid *ProDgnsLTP*–*DgnsLTP*‐*LUC* was obtained. The above recombinant plasmids were all constructed by Sangon Biotech Company.

For the LCI experiments, the abovementioned related recombinant plasmids were first cotransformed with *Agrobacterium* GV3101 and then transiently expressed in tobacco leaves. Then, the kit instructions for detecting firefly enzymes were followed, and a live imaging instrument was used to perform fluorescence detection.

To measure double luciferase, samples were collected from the leaves after transient expression and then analysed on a microplate reader according to the instructions of the Dual Luciferase Reporter Gene Assay kit (Beyotime, China).

### Determination of hydrogen peroxide (H_2_O_2_) and superoxide anion (O_2_^‐^) and NBT and DAB staining

Measurement of the H_2_O_2_ and O_2_^‐^ contents was performed according to the steps of a quantitative measurement kit (Suzhou Keming Biological Co., Ltd., Suzhou, China), and NBT and DAB were used for histochemical staining according to a previously reported method (Wang *et al*., 2017).

### POD, APX, CAT activity, malondialdehyde content and relative electrolyte leakage

The POD, APX and CAT activities of the samples from the low temperature treatments with different time gradients (4°C for 0 h, 3 h, 6 h, 12 h and 24 h, and 75% relative humidity) were tested according to the kit instructions (Nanjing Jiancheng, China), with three replicates per sample. Analyses of the malondialdehyde content and relative electrolyte leakage followed previously reported methods (Gilmour *et al*., 1998; Kjellsen *et al*., 2010).

## Conflicts of interest statement

The authors declare no competing interests.

## Author contributions

Q.H. designed the experiments, conducted all the data analysis and wrote the manuscript; X.L. and X.Y. performed the experiments, analysed the data and wrote the manuscript; Y.L., P.L., Q.Z. and H.B. performed the experiments and analysed the data; B.J., Y.P., F.Z., L.Z. and Y.J. analysed the data. Q.L. designed the experiment, conceived the project and supervised the study. All authors approved the final version of the manuscript.

## Supporting information


**Figure S1** Phylogenetic analysis and sequence alignment of the DgTIL1 protein with known homologs in other plants.
**Figure S2** Phylogenetic analysis and sequence alignment of the DgnsLTP protein with nsLTP protein from different species.
**Figure S3** Analysis of the degradation of DgnsLTP protein in chrysanthemum.
**Figure S4** DgTIL1 protein expression and modification.
**Figure S5** Comparison of APX and CAT activity in the WT lines and transgenic chrysanthemum.
**Table S1** Cold‐responsive TILs genes identified from a cold stress transcriptome analyses.
**Table S2** Screening the potential interacting proteins of DgTIL1.
**Table S3** Primers used for expression analysis.
