# Supplementary material for: Lysine crotonylation of DgTIL1 at K72 modulates cold tolerance by enhancing DgnsLTP stability in chrysanthemum
Source: Plant Biotechnol J. 2021 Jan 21;19(6):1125–40. doi: 10.1111/pbi.13533 (PMC8196654; doi:10.1111/pbi.13533)
Supplement: Supplementary file 1 — Figure S1 Phylogenetic analysis and sequence alignment of the DgTIL1 protein with known homologs in other plants. Figure S2 Phylogenetic analysis and sequence alignment of the DgnsLTP protein with nsLTP protein from different species. Figure S3 Analysis of the degradation of DgnsLTP protein in chrysanthemum. Figure S4 DgTIL1 protein expression and modification. Figure S5 Comparison of APX and CAT activity in the WT lines and transgenic chrysanthemum. Table S1 Cold‐responsive TILs genes identified from a cold stress transcriptome analyses. Table S2 Screening the potential interacting proteins of DgTIL1. Table S3 Primers used for expression analysis. [file PBI-19-1125-s001.zip › pbi13533-sup-0006-TableS1-S3-1.docx]

**Supplemental Table**

**Supplemental Table 1.** Cold-responsive *TILs* genes identified from a cold stress transcriptome analyses using cDNA samples extracted from using cold-treated(4°C for 24 h and -4°C for 4 h) and non-treated(temperature, 25°C) chrysanthemum seedlings.The significantly up-regulated (> 0 fold) and down-regulated (< 0 fold) ,*TILs* genes after cold treatment are listed.

| **Gene_id** | **Description** | **log2ratio(TvsCK)** |
| --- | --- | --- |
| Cluster-2961.171421 | PREDICTED: apolipoprotein D-like [Cucumis melo]>gi\|659070937\|ref\|XP_008457334.1\| PREDICTED: apolipoprotein D-like [Cucumis melo] ,Nicotiana tabacum temperature-induced lipocalin (TIL) mRNA | 4.2127 |
| Cluster-2961.190245 | temperature-induced lipocalin [Nicotiana tabacum], Solanum tuberosum temperature-induced lipocalin (TIL) mRNA | 3.0244 |
| Cluster-2961.124360 | PREDICTED: apolipoprotein D-like [Cucumis melo]>gi\|659070937\|ref\|XP_008457334.1\| PREDICTED: apolipoprotein D-like [Cucumis melo], Nicotiana tabacum temperature-induced lipocalin (TIL) mRNA, complete cds | 3.1498  Up |
| Cluster-2961.124362 | PREDICTED: apolipoprotein D-like [Cucumis melo]>gi\|659070937\|ref\|XP_008457334.1\| PREDICTED: apolipoprotein D-like [Cucumis melo], Nicotiana tabacum temperature-induced lipocalin (TIL) mRNA, complete cds | 2.9332 |
| Cluster-2961.35585 | temperature-induced lipocalin [Populus tremuloides], Solanum lycopersicum chromosome ch12, complete genome | 0.9855 |
| Cluster-2961.178928 | PREDICTED: apolipoprotein D-like [Pyrus x bretschneideri], Nicotiana tabacum temperature-induced lipocalin (TIL) mRNA | -0.27378 |
| Cluster-2961.145653 | temperature-induced lipocalin' [Solanum lycopersicum]>gi\|77744859\|gb\|ABB02383.1\| temperature-induced lipocalin' [Solanum lycopersicum], PREDICTED: Solanum tuberosum apolipoprotein D-like (LOC102588695), transcript variant X2, mRNA | -0.14158  Down |
| Cluster-2961.250416 | temperature-induced lipocalin' [Populus balsamifera]>gi\|209967465\|gb\|ACJ02357.1\| temperature-induced lipocalin [Populus tremula x Populus alba] | -0.82598 |
|  |  |  |
|  |  |  |

**Supplemental Table 2.** Screening the potential interacting proteins of DgTIL1.

Ubiquitin membrane yeast two-hybrid assays were performed to identify the interacting proteins of DgTIL1 using pBT3-N-DgTIL1 as the bait. The cDNA fragments of positive yeast strains were identified by sequencing. As a result, 10 potential interacting proteins were obtained including XP_022040976.1, PWA73340.1, XP_021993551.1, PWA82850.1, PWA47841.1, XP_023769050.1, PWA74152.1, PWA99194.1, PWA74347.1 and PWA87346.1.

| **gene bank** | **gene name** |  |
| --- | --- | --- |
| XP_022040976.1 | membrane protein of ER body-like protein isoform X3 [Helianthus annuus] |  |
| PWA73340.1 | RNA-binding S4 domain-containing protein [Artemisia annua] |  |
| XP_021993551.1 | - non-specific lipid-transfer protein-like protein At5g64080 [Helianthus annuus] |  |
| PWA82850.1 | C2 calcium-dependent membrane targeting [Artemisia annua] |  |
| PWA47841.1 | hypothetical protein CTI12_AA495870 [Artemisia annua] |  |
| XP_023769050.1 | dnaJ homolog subfamily C member 2 [Lactuca sativa] |  |
| PWA74152.1 | hypothetical protein CTI12_AA255390 [Artemisia annua] |  |
| PWA99194.1 | chlorophyll A-B binding protein [Artemisia annua] |  |
| PWA74347.1 | CAAX amino terminal protease [Artemisia annua] |  |
| PWA87346.1 | 30S ribosomal protein S1 protein [Artemisia annua] |  |

**Supplemental Table 3.** Primers used for expression analysis

| Primer | Function | | Sequence(5'-3') |
| --- | --- | --- | --- |
| *EF1α-1*  *EF1α-2*  T-1  T-2  T-3  T-4  T^R^-5  T^R^-6  T^N^-7  T^N^-8  L-1  L-2  L-3  L-4  P-1  P-2  TIL1-5  TIL1-6  NsLTP-5  NsLTP-6 | | Internal reference primer  Internal reference primer  PCR amplification primers  PCR amplification primers  qRT-PCR primers  qRT-PCR primers  qRT-PCR primers  qRT-PCR primers  qRT-PCR primers  qRT-PCR primers  PCR amplification primers  PCR amplification primers  qRT-PCR primers  qRT-PCR primers  qRT-PCR primers  qRT-PCR primers  Natural promoter primer  Natural promoter primer  Natural promoter primer  Natural promoter primer | F：GACGGCATCTCTAGAGGCCA  R：CAGTCAACTTCCGGTCACCG  F：AGTAAAGGGTGTTGATCTAGCAAG  R：AGAGTGACTTGATATACCACACACC  F：CTAGCAAGGTACATGGGGAGA  R：AACCCAATAATCACCAGTCACA  F：TCCATCAAGGTTTCAGCCCA  R：AATAGGCAAAAACGGTGGC  F：TCTAGCAAGGTACATGGGGA  R：GGTGGCACCCAAAACTTAA  F：CTTAACCACCCTCCTCCTCC  R：AAACAGAGCCAATCGACATGTAT  F：TGATGCAGAGTGTTTGTGTGAA  R：GATGAGCTAGAGGCTTGGGAC  F：TTTGGTTCCCATTGTTGTGTT  R：ATCACAACCCTGAACGAAACA  F：ACTCATGATGGATAAGCATGCA  R：GTTTTGTGAGTTATGAGGTGGGA  F：CCCAAAAAATAGAGGCTCTGTAC  R：TTTGGATGTTTTAGTGTGTGTGA |
