# Supplementary figures and images for: Lysine crotonylation of DgTIL1 at K72 modulates cold tolerance by enhancing DgnsLTP stability in chrysanthemum
Source: Plant Biotechnol J. 2021 Jan 21;19(6):1125–40. doi: 10.1111/pbi.13533 (PMC8196654; doi:10.1111/pbi.13533)

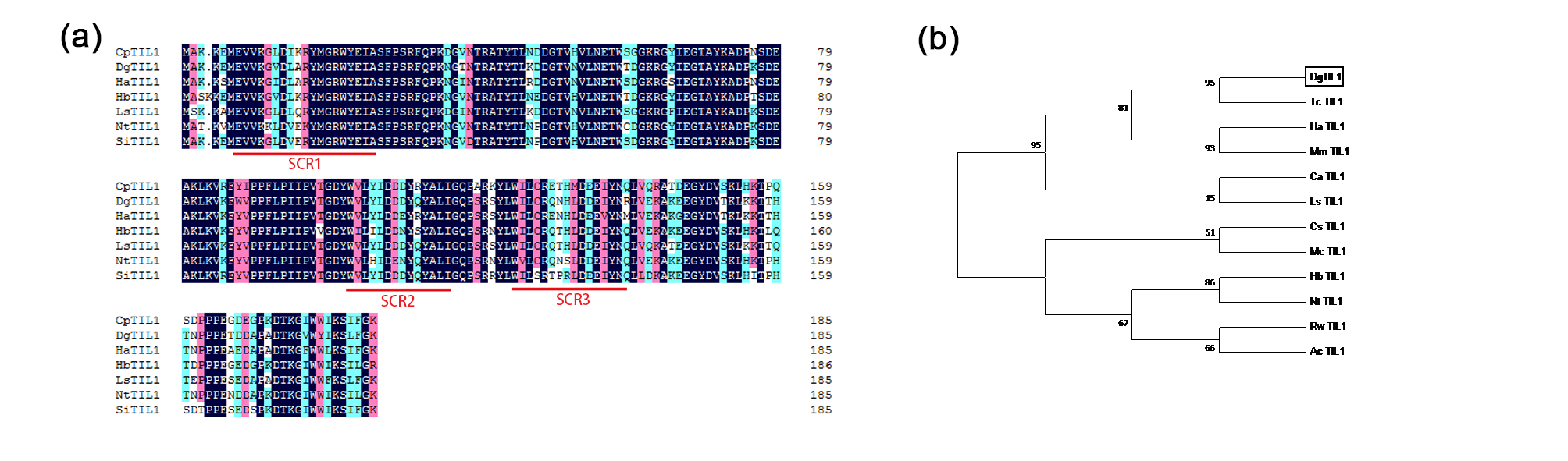

Supplement: Supplementary file 1 — Figure S1 Phylogenetic analysis and sequence alignment of the DgTIL1 protein with known homologs in other plants. Figure S2 Phylogenetic analysis and sequence alignment of the DgnsLTP protein with nsLTP protein from different species. Figure S3 Analysis of the degradation of DgnsLTP protein in chrysanthemum. Figure S4 DgTIL1 protein expression and modification. Figure S5 Comparison of APX and CAT activity in the WT lines and transgenic chrysanthemum. Table S1 Cold‐responsive TILs genes identified from a cold stress transcriptome analyses. Table S2 Screening the potential interacting proteins of DgTIL1. Table S3 Primers used for expression analysis. [file PBI-19-1125-s001.zip › pbi13533-sup-0001-FigS1-1.tif]

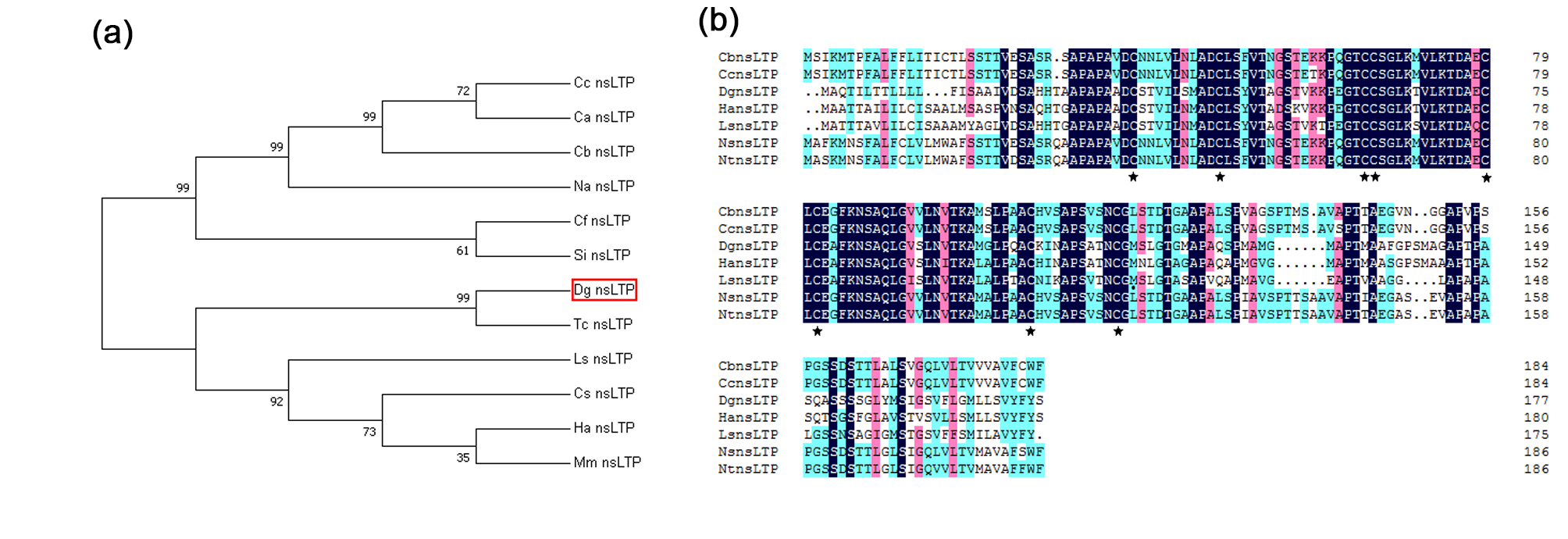

Supplement: Supplementary file 1 — Figure S1 Phylogenetic analysis and sequence alignment of the DgTIL1 protein with known homologs in other plants. Figure S2 Phylogenetic analysis and sequence alignment of the DgnsLTP protein with nsLTP protein from different species. Figure S3 Analysis of the degradation of DgnsLTP protein in chrysanthemum. Figure S4 DgTIL1 protein expression and modification. Figure S5 Comparison of APX and CAT activity in the WT lines and transgenic chrysanthemum. Table S1 Cold‐responsive TILs genes identified from a cold stress transcriptome analyses. Table S2 Screening the potential interacting proteins of DgTIL1. Table S3 Primers used for expression analysis. [file PBI-19-1125-s001.zip › pbi13533-sup-0002-FigS2-1.tif]

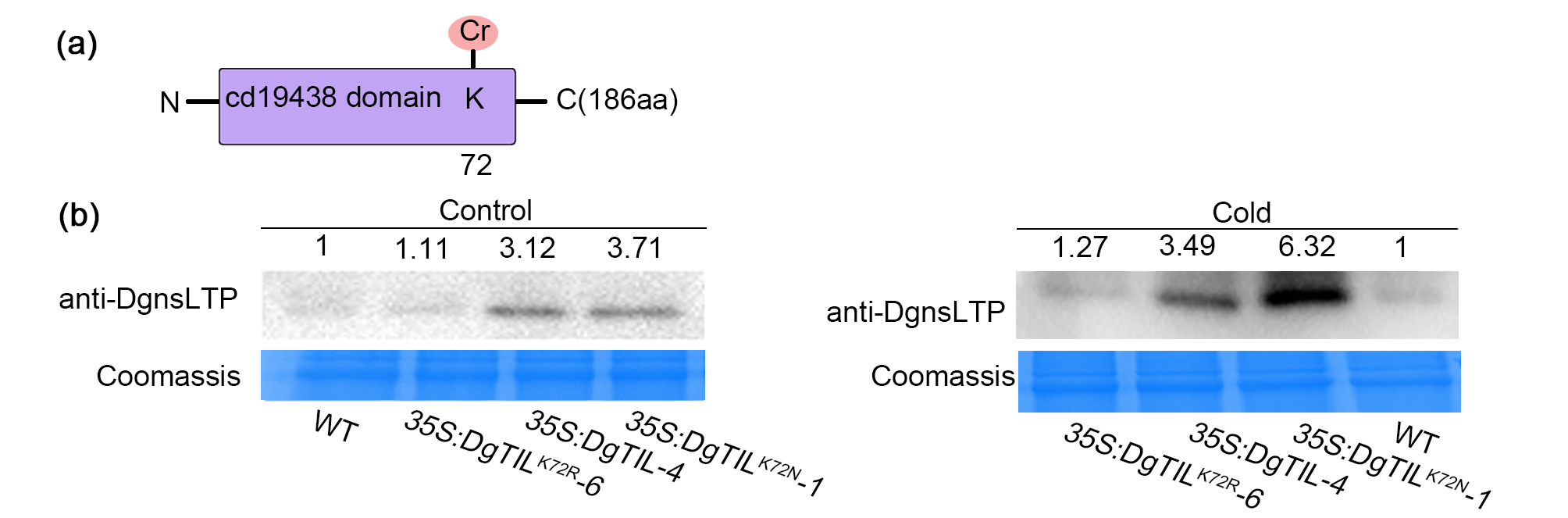

Supplement: Supplementary file 1 — Figure S1 Phylogenetic analysis and sequence alignment of the DgTIL1 protein with known homologs in other plants. Figure S2 Phylogenetic analysis and sequence alignment of the DgnsLTP protein with nsLTP protein from different species. Figure S3 Analysis of the degradation of DgnsLTP protein in chrysanthemum. Figure S4 DgTIL1 protein expression and modification. Figure S5 Comparison of APX and CAT activity in the WT lines and transgenic chrysanthemum. Table S1 Cold‐responsive TILs genes identified from a cold stress transcriptome analyses. Table S2 Screening the potential interacting proteins of DgTIL1. Table S3 Primers used for expression analysis. [file PBI-19-1125-s001.zip › pbi13533-sup-0003-FigS3-1.tif]

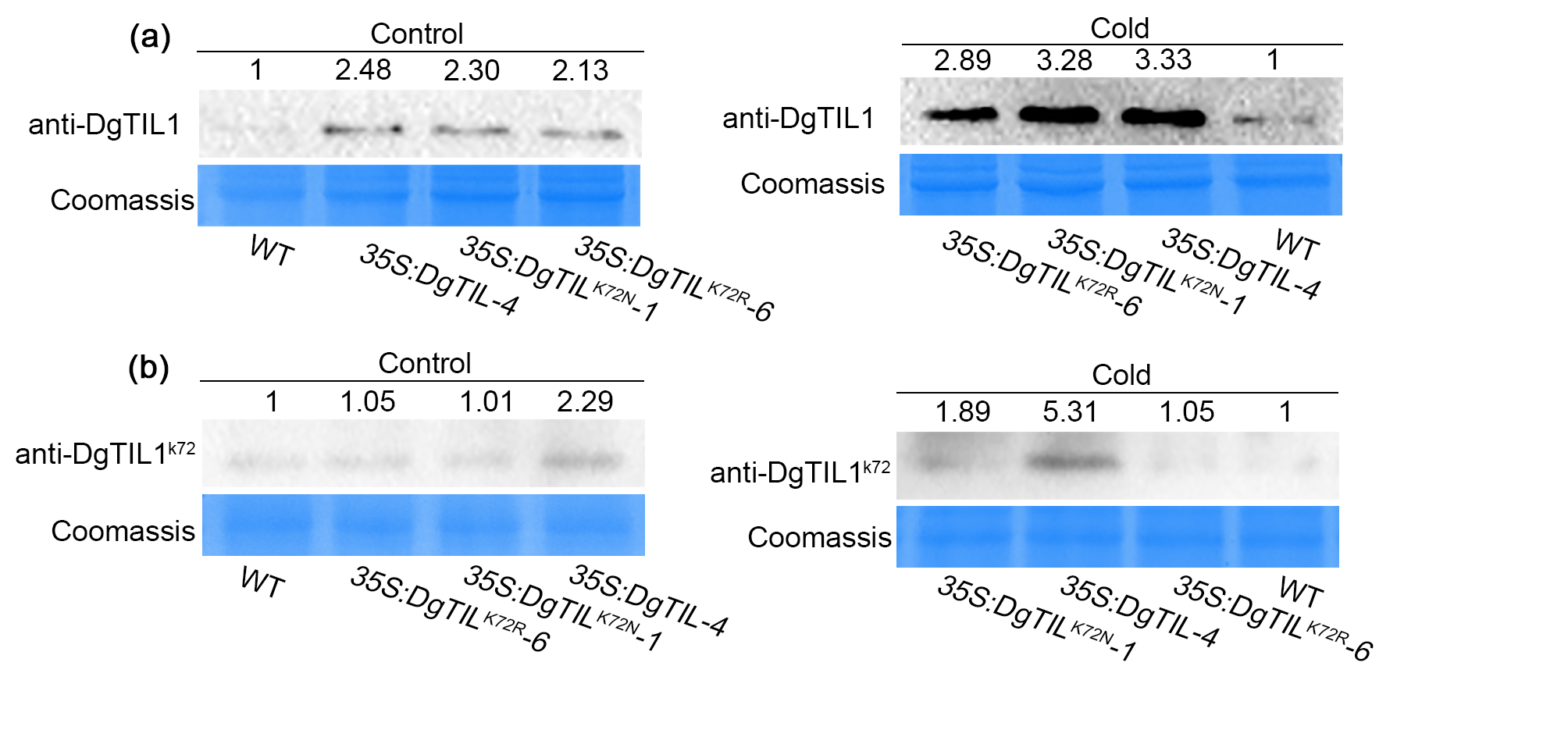

Supplement: Supplementary file 1 — Figure S1 Phylogenetic analysis and sequence alignment of the DgTIL1 protein with known homologs in other plants. Figure S2 Phylogenetic analysis and sequence alignment of the DgnsLTP protein with nsLTP protein from different species. Figure S3 Analysis of the degradation of DgnsLTP protein in chrysanthemum. Figure S4 DgTIL1 protein expression and modification. Figure S5 Comparison of APX and CAT activity in the WT lines and transgenic chrysanthemum. Table S1 Cold‐responsive TILs genes identified from a cold stress transcriptome analyses. Table S2 Screening the potential interacting proteins of DgTIL1. Table S3 Primers used for expression analysis. [file PBI-19-1125-s001.zip › pbi13533-sup-0004-FigS4-1.tif]

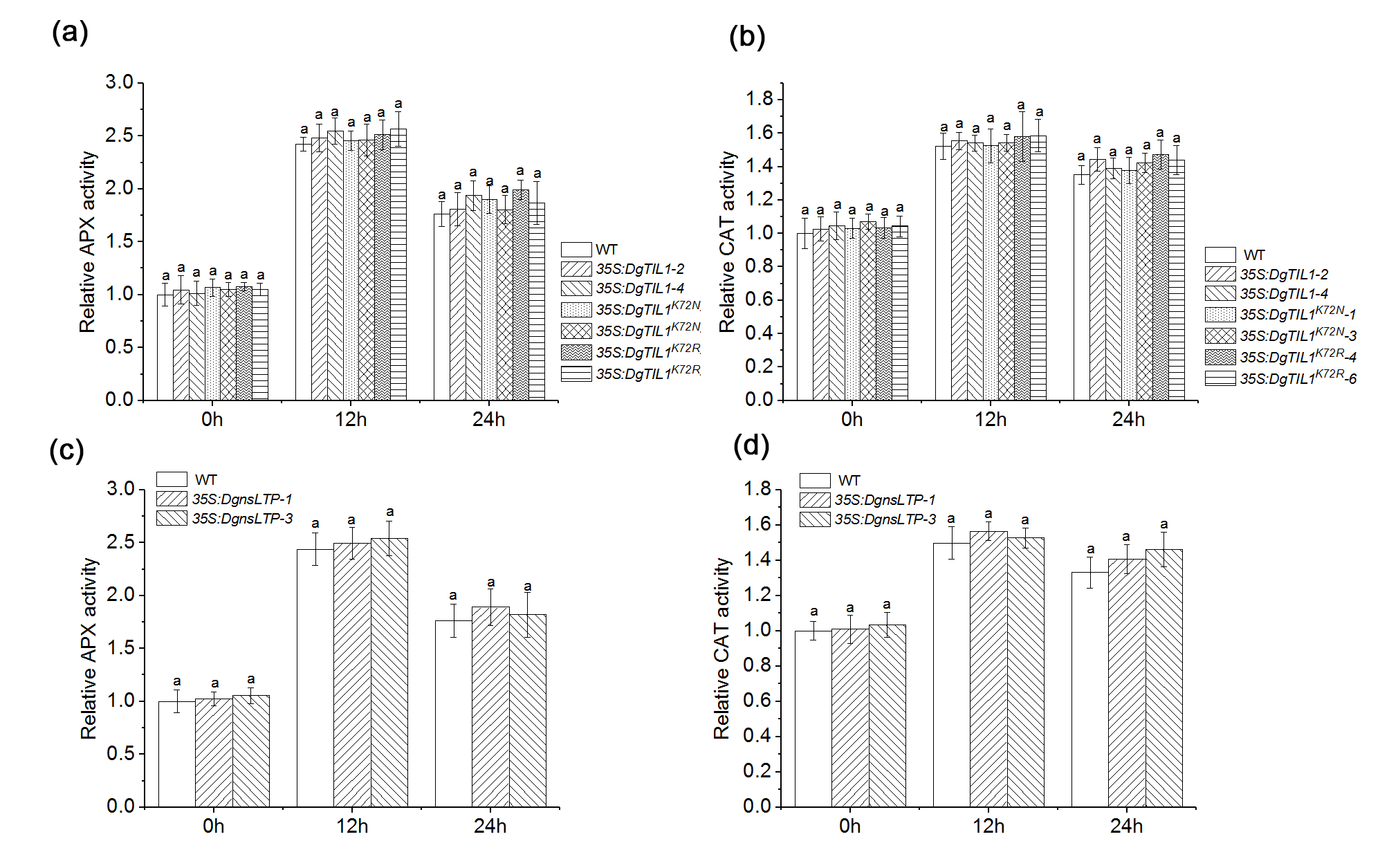

Supplement: Supplementary file 1 — Figure S1 Phylogenetic analysis and sequence alignment of the DgTIL1 protein with known homologs in other plants. Figure S2 Phylogenetic analysis and sequence alignment of the DgnsLTP protein with nsLTP protein from different species. Figure S3 Analysis of the degradation of DgnsLTP protein in chrysanthemum. Figure S4 DgTIL1 protein expression and modification. Figure S5 Comparison of APX and CAT activity in the WT lines and transgenic chrysanthemum. Table S1 Cold‐responsive TILs genes identified from a cold stress transcriptome analyses. Table S2 Screening the potential interacting proteins of DgTIL1. Table S3 Primers used for expression analysis. [file PBI-19-1125-s001.zip › pbi13533-sup-0005-FigS5-1.tif]
